# Genomic diversity and virulence of Pasteurella multocida in Norwegian calves

**DOI:** 10.1099/mgen.0.001735

**Published:** 2026-06-08

**Authors:** Asal Ahmadi, Veslemøy Sunniva Oma, Lise Marie Ånestad, Silje Enge Falkeid, Amulya Baral, Abdolrahman Khezri, Thea Blystad Klem, Maria Stokstad, Ann-Katrin Llarena

**Affiliations:** 1 Department of Paraclinical Sciences, Faculty of Veterinary Medicine, Norwegian University of Life Sciences, Ås, Norway; 2 Department of Production Animal Clinical Sciences, Faculty of Veterinary Medicine, Norwegian University of Life Sciences, Ås, Norway; 3 Department of Animal Health, Welfare and Food Safety, Veterinary Institute, Ås, Norway; 4 Department of Biotechnology, Faculty of Ecology, Agriculture, and Biotechnology, University of Inland Norway, Hamar, Norway

**Keywords:** bovine respiratory disease complex, *Pasteurella multocida*, population genomics, intra-animal differences, transmission dynamics, virulence factors

## Abstract

The bovine respiratory disease (BRD) complex is a major health and welfare challenge in the cattle industry worldwide, with substantial economic and antimicrobial resistance implications. *Pasteurella multocida* (PM) is a key bacterial agent of BRD; however, knowledge gaps remain regarding its transmission dynamics, genomic diversity and virulence-associated gene repertoire in cattle. In this cross-sectional study, we used population genomics to characterize PM isolates circulating in Norwegian calves and compare them with global bovine PM lineages. By integrating genomics with epidemiological and clinical data from both healthy and BRD-affected animals across different herd production systems, we identified associations between PM genotypes, herd-level factors and disease status. Fattening herds harboured greater PM diversity than dairy herds, and herd-specific population structures were common, with limited evidence of recent inter-herd transmission. Sequence type (ST) 173 was strongly associated with clinical signs of respiratory disease and exhibited a distinct genomic profile, including genes linked to iron acquisition and quorum sensing. A possible tropism for the lower respiratory tract was also observed for this ST. In contrast, ST79 was more prevalent among healthy animals and appeared to act predominantly as a commensal. Nasal swabs often, but not always, reflected the PM strain present in the lungs, highlighting both the utility and limitations of non-invasive diagnostics. Collectively, these findings demonstrate that herd management and bacterial genetic diversity play key roles in BRD epidemiology. Accordingly, targeted control strategies should account for the heterogeneity and pathogenic potential of circulating PM strains.

**Impact Statement**

*Pasteurella multocida* is a major bacterial contributor to bovine respiratory disease, a condition with substantial animal welfare and economic consequences for the cattle industry. Despite its importance, genomic data describing the population structure, strain-level pathogenicity, transmission dynamics and virulence-associated diversity of *P. multocida* circulating in cattle are scarce. Also, it remains unclear whether upper respiratory tract samples reliably represent pathogenic lineages present in the lower respiratory tract. Here, population genomics was integrated with epidemiological and clinical metadata to characterize *P. multocida* isolates from healthy and diseased calves across different Norwegian herd production systems, with comparisons to global bovine lineages. These findings provide a genomic framework for understanding herd-level transmission patterns, accessory genome content (virulence- and antimicrobial resistance–associated genes), genotype–disease associations and sampling representativeness. Such studies could establish a baseline for future genomic surveillance of *P. multocida* in cattle and support the development of more targeted approaches to controlling bovine respiratory disease.

## Data Summary

All whole-genome sequences of *Pasteurella multocida* isolates generated in this study, alongside associated metadata, were deposited in the European Nucleotide Archive under accession number PRJEB106488. Isolate-specific information for Norwegian isolates is provided in the Supplementary Material (Norwegian Metadata-Dataset 1). Comparative genomic analyses also included publicly available bovine *P. multocida* genomes (National Center of Biotechnology Information Metadata). The authors confirm that all supporting data and protocols are provided within the article or in the Supplementary Material.

## Introduction

Bovine respiratory tract infection/disease complex (BRD) is one of the most significant health and economic challenges in the cattle industry globally, causing over $3 billion annual losses through reduced productivity, treatment expenses and mortality in both beef and dairy sectors [1,2]. Beyond its economic impact, BRD leads to considerable animal suffering by compromising multiple welfare domains, including physical health, functional behaviours and affective state [2,4]. Furthermore, BRD contributes to the emergence of antimicrobial resistance (AMR), raising public health concerns regarding the potential transmission of resistant bacteria along the food chain [5].

As a multifactorial and polymicrobial complex, BRD progression and outcome are shaped by environmental, management and host-related factors that compromise immunity and increase susceptibility to infectious agents. These factors often interact synergistically, resulting in herd-level outbreaks [6,8]. Within this context, viral pathogens frequently impair respiratory defences and promote secondary bacterial infections. However, the distinction between primary and secondary pathogens in BRD is not clear-cut and may vary between outbreaks and individual cases [9]. Among bacterial agents, *Pasteurella multocida* (PM) is recognized as a major contributor to BRD [10,12].

This Gram-negative bacterium is associated with disease manifestations ranging from subclinical carriage to severe multisystemic infections in mammals, domesticated birds and humans. According to the World Organization for Animal Health, ~20% of bovine mortality worldwide is attributed to *Pasteurella* [13]. Among the subspecies *multocida*, *gallicida* and *septica*, the subspecies *multocida* is most commonly linked to BRD [11,14]. PM is a normal commensal of the upper respiratory tract but can become opportunistically pathogenic during host stress, colonizing the lower airways and causing BRD. In contrast to BRD-associated strains, genetically and phenotypically distinct strains of PM cause other diseases, such as haemorrhagic septicaemia in cattle and other bovines [12,15, 16].

PM exhibits extensive genetic and phenotypic diversity. It is classified into five capsular serogroups (A, B, D, E and F) and 16 LPS serovars, which are further grouped into eight LPS types using a multiplex PCR assay [17]. In BRD, the most common type globally is capsular type A, LPS group 3 and sequence type (ST) 79 [18,19].

PM’s pathogenicity is mediated by a diverse arsenal of virulence factors (VFs), including capsule, LPS, iron-acquisition systems, outer membrane proteins (OMPs), adhesins and secreted enzymes. Together, these factors facilitate host colonization, immune evasion and tissue damage. Adhesins such as PtfA, FimA, TadD, Flp1/2, Hsf-1/2 and PfhB1/2 promote mucosal adherence [20,21], while OmpA and iron-binding proteins, including HgbA and TbpA, contribute to host invasion through binding to fibronectin and other extracellular matrix components [11,22]. The capsule protects against phagocytosis and complement-mediated killing, supporting intracellular survival [11,13, 20, 23]. LPS triggers the release of inflammatory cytokines, contributing to lung pathology [10,11]. In addition, sialidases (NanH and NanB) enhance virulence by exposing host cell receptors, disrupting mucosal barriers and modulating host immune signalling [20].

Despite extensive research on pasteurellosis, major gaps remain in our understanding of the epidemiology and pathogenic diversity of PM strains in cattle. Transmission dynamics between and within herds are poorly defined, despite their importance for controlling the spread of BRD. Intra-animal strain diversity is also insufficiently characterized, complicating the interpretation of bacterial diagnostics from samples collected at different anatomical sites within the respiratory tract. Moreover, it is unclear whether specific strains possess genetic traits that promote transmission, persistence or host adaptation – knowledge that is critical for effective herd management. Finally, the VFs required for PM to cause BRD have not been fully resolved, and it is unknown whether all strains circulating in cattle have the potential to cause disease.

Therefore, the main objective of this study was to investigate the population structure, genomic diversity and VF repertoire of PM circulating in Norwegian calves and to compare these findings with global bovine PM lineages. The specific aims were as follows: (i) identify associations between PM genotypes, herd-level factors and disease status by integrating PM population genomics with epidemiological and clinical data from both healthy and BRD-affected calves across different herd production systems; (ii) assess the representativeness of different respiratory sampling methods for detecting pathogenic lineages; (iii) explore the transmission dynamics of PM between and within herds and individuals in Norwegian cattle populations.

## Methods

### Bacterial isolates and study population

A total of 370 PM isolates were included in this study. The isolates were collected between 2021 and 2022 as part of a larger study on BRD [24]. They originated from respiratory tract samples taken from 149 calves across 15 herds, including both dairy and fattening herds, with a history of BRD in the mid and southern regions of Norway. In the Norwegian cattle production system, ‘fattening herds’ are specialized operations that purchase young calves (primarily dairy bull calves) after weaning and rear them for beef production until slaughter. These systems are generally smaller and less intensive than feedlot operations in other countries. All herds included were larger than the national average herd size in 2020 and had a recurring history of BRD [24].

In each herd, 10–20 calves were sampled once. The respiratory health of selected calves was assessed using a modified version of the clinical scoring system developed by [25]. Here, respiratory rate, rectal temperature, nasal discharge, coughing, lung auscultation and general demeanour were assessed. Each parameter contributed 0–3 points, resulting in a total clinical score ranging from 0 to 18. Calves with a total score of ≤5 were classified as healthy (*n*=58), while those >5 were classified as sick (*n*=91).

Nasal swabs (N), guarded nasopharyngeal swabs (D) and non-endoscopic bronchoalveolar lavage (BAL) samples were collected from each calf (Table 1). Following culturing on 5% bovine blood agar supplemented with V-factor, 1 to 12 colonies per animal with morphology consistent with PM – typically small, greyish, non-haemolytic – were selected for further analysis (see the Supplementary Material for additional information; Table S1, available in the online Supplementary Material). Calves (*n*=58) from which at least one PM isolate was obtained from each of the three sampling sites (N, D and BAL) were defined as ‘triple-site calves’. These isolates (*n*=227) were used to evaluate site-specific and intra-individual diversity of PM (Triple-site-isolate-Dataset).

**Table 1.** Overview of PM isolates recovered from N, D and BAL samples from Norwegian calves with and without BRD, adapted from [24]

			**Herds**				
			**Dairy**		Fattening*		**Total**
**PM detection in Norwegian herds**			8		7		15
			**Health status**				
			**Healthy**	**BRD**	**Healthy**	**BRD**	**Total**
**Calves with PM detection**			40	38	18	53	149
**PM isolates**			84	115	39	132	370
**Sampling method**	**N**	**PM isolates/calves with PM detection**	30/30	44/32†	15/12†	34/28†	123/102†
	**D**	**PM isolates/calves with PM detection**	29/27†	39/32†	12/12	43/31†	123/102†
	**BAL**	**PM isolates/calves with PM detection**	25/25	32/23†	12/10†	55/45†	124/103†
**Streptomycin-resistant isolates (AST)/calves with resistant PM detection**			78/38	110/35	19/10	46/28‡	253/111†

*‘Fattening herds’ refers to Norwegian production units that purchase and raise weaned calves for beef until slaughter, analogous but not identical to ‘feedlot cattle’ systems in other countries.

†The number of PM isolates exceeds the number of calves due to the inclusion of multiple isolates from individual animals. **AST:** antibacterial susceptibility testing results for PM isolates. The antimicrobial panel tested in [24] included penicillin, amoxicillin-clavulanic acid, trimethoprim-sulfamethoxazole, tetracycline, enrofloxacin, florfenicol and streptomycin.

‡One isolate in this category exhibited resistance to tetracycline in addition to streptomycin.

Furthermore, 345 publicly available PM genomes, tagged with 'Bovine' and equivalent host terms (search terms: Bovine and/or bovine and/or Cattle and/or cattle and/or Beef cattle and/or Bos taurus and/or Bos taurus linnaeus and/or Buffalo and/or buffalo and/or buffalo calf and/or Calf and/or calf and/or Bubalus bubalis and/or Bison and/or Bison bison and/or Cow and/or cow), were downloaded from the National Center of Biotechnology Information (NCBI) Pathogen Detection browser for comparative analysis with detailed metadata (13 December 2024, NCBI Metadata).

### DNA isolation

The PM cryo-cultures were stored at −80 °C until the day of the experiment, after which a loop of frozen material was plated on 5% bovine blood nutrient agar (Norwegian Veterinary Institute, Ås, Norway). The plates were incubated aerobically at 37 °C with 5% CO_2_ for 24 h. Single colonies were then inoculated in 2 ml brain and heart infusion broth (Norwegian Veterinary Institute) and incubated for 18 h at 37 °C with shaking at 200 r.p.m. DNA was subsequently extracted and purified from 500 µl aliquots using the DNeasy Blood and Tissue Isolation Kit (Qiagen, MD, USA), according to the manufacturer’s instructions.

### Whole-genome sequencing

DNA was quantified using the dsDNA broad range assay (Thermo Fisher Scientific, MA, USA) on a Qubit 4 Fluorometer (Thermo Fisher Scientific, MA, USA). DNA quality was fluorometrically assessed using mySPEC (VWR Avantor, Leicestershire, UK) and subjected to library preparation with the Illumina DNA prep kit (Illumina, CA, USA) according to the manufacturer’s protocols. Sequencing was performed using Illumina technology on either a NextSeq (2×150 paired-end reads) or a MiSeq (2×300 paired-end reads) platform at the Norwegian Veterinary Institute, Ås, Norway.

### Bioinformatics analysis

The bioinformatic analysis followed the workflow outlined in Fig. S1 in the Supplementary Material. Detailed procedures for read trimming, quality control, genome assembly and quality assessment, species confirmation and genome annotation are described in the Supplementary Material.

### Subspecies assignment, capsule/LPS genotyping, MLST and core genome MLST

Subspecies assignment was done by comparing the average nucleotide identity (ANI) between the assemblies and reference type strain (ANI threshold ≥98%) for subspecies *multocida* NCTC 10322 (Accession: LT906458), *gallicida* NCTC 10204 (Accession: LR134298) and *septica* NCTC 11995 (Accession: UBSV00000000). Differentiation between subsp. *multocida* and subsp. *gallicida* was further studied based on the detection of the *gatD* gene as described by [26,27].

The capsular and LPS loci of assembled genomes were identified using BLASTn [28] against a custom database downloaded from the online server ivsmlst.sund.ku.dk that contains nucleotide sequences coding for five different capsular serotypes and eight LPS serogroups [29]. Hits with the highest percentage of identity (≥99%), bit score (≥1,000) and coverage over 50% (alignment length/subject length) were assigned as the corresponding capsular and LPS serotypes for each assembly.

STs were assigned using multi-locus sequence typing (MLST) v2.23.0 [30] with the Rural Industries Research and Development Corporation (RIRDC) scheme available from the pubMLST website (https://pubmlst.org/) [31]. RIRDC-MLST numbers for newly identified STs were acquired by submitting assemblies to the pubMLST database.

### AMR, VFs and mobile genetic element identification

To assess the pathogenicity and genomic diversity of the PM isolates, assemblies were screened for both acquired antimicrobial resistance genes (ARGs) and resistance-associated mutations, VFs, plasmids and integrative and conjugative elements (ICEs). Sequence similarity searches were performed using BLASTn and tBLASTn against curated databases (Table 2). Only hits meeting the sequence identity and coverage thresholds specified in Table 2 were retained.

**Table 2.** blast parameters and databases used to detect ARGs, VFs, plasmids and ICEs in PM genomes

Target	Query	Subject	blast suite	Databases	Thresholds
**ARG**	Assembly contigs	AMR gene database	BLASTn	Merged and de-replicated genes from Megares v3.0 [79], CARD v4.0.0 [80] and ResFinder v4.7.2 [81]	PI ≥80%; Cov (len/slen) ≥50%
**VF**	Virulence protein sequences	Assembly contigs	tBLASTn	Core VFDB [82]; PM virulence protein sequences downloaded from NCBI and VFs previously reported by [19,83]	PI ≥80%; Cov (len/qlen) ≥50%
**Plasmid**	Plasmid assembly contigs	PLSDB plasmid sequences	BLASTn	PLSDB [84]	PI ≥80%; Cov (len/slen) ≥50%
**ICEPmu1** **ICE**	Assembly contigs ICE proteins	ICEPmu1 sequence Assembly contigs	BLASTn tBLASTn	ICEberg 2.0 [85]	PI ≥80%; Cov (len/slen) ≥50% PI ≥80%; Cov (len/qlen) ≥50%

Cov, coverage; len, alignment length; PI, percentage identity; qlen, query length; slen, subject length.

### Population genomics

Phylogenetic analyses were performed on two datasets: (i) assemblies generated in this study (Norwegian Metadata-Dataset 1) and (ii) Dataset 1 combined with publicly available bovine PM assemblies (collectively Norwegian-NCBI-Dataset 2). The pan- and core-genomes were defined, and core-gene alignments were generated for both datasets using Panaroo v1.5.1.4, strict mode [32]. As recombination is limited in PM [33], phylogenetic inference was performed on the core-gene alignment phylogeny using the maximum likelihood method implemented in IQ-tree v2.3.6 [34]. Resulting phylogenies were visualized by the Interactive Tree of Life (iTOL) v7.0, available at https://itol.embl.de/ [35].

To investigate potential transmission between and within herds and compare sampling sites within individual animals, clonal relationships among the Norwegian isolates (Norwegian Metadata-Dataset 1) were assessed using a core genome MLST (cgMLST) scheme derived from whole-genome MLST (wgMLST). Loci present in ≥95% of genomes were included (cgMLST95), and schema creation and allele calling were performed using the chewBBACA suite [36]. Isolate relationships were visualized using a minimum spanning tree (MST) constructed with the goeBURST algorithm implemented in Phyloviz v. 2 [37]. Hierarchical clustering of cgMLST95 allelic profiles was conducted in ReporTree v2.6.0 [38] with a threshold of ≤5 allelic differences calculated by ReporTree to define clusters; isolates within the same ReporTree cluster were considered clonal.

To identify putative VFs associated with disease development, a genome-wide association study (GWAS) was performed using Scoary v1.6.16 [39] on Norwegian Metadata-Dataset 1. The analysis was based on Panaroo-generated gene presence–absence matrices and assessed associations with specific traits, including clinical status and ST. Candidate genes were considered uniquely associated with a given trait if both sensitivity and specificity were 100%, whereas genes with ≥85% sensitivity and specificity for the trait were considered overrepresented. Corresponding protein sequences to candidate genes were retrieved from the Panaroo output and functionally annotated using eggNOG-mapper v2.0 [40]. The resulting annotations were processed with TBtools-II v2.2 [41] for Windows to extract Clusters of Orthologous Groups (COG) codes and KEGG (Kyoto Encyclopedia of Genes and Genomes) Orthology (KO) identifiers. Finally, COG functional categories were assigned, and KO identifiers were subsequently submitted to the KEGG Mapper v5 online platform [42] to infer associated metabolic pathways.

### Statistics

A smaller dataset (Dataset 3) was created by collapsing isolates from the same calf into a single representative when assemblies differed by ≤5 cgMLST alleles and shared identical virulence and AMR profiles. This stringent clonality criterion was applied to ensure clinical relevance of the sampling methodology. Dataset 3 was used to assess the following: (i) intra-animal diversity of PM, (ii) ST diversity across herds and production systems using Shannon’s diversity index (H) calculated in PAST software v4.03 for Windows [43] and (iii) associations between disease status (response variable) and predictors (fixed effects), including STs, VFs and production systems. Associations were tested using a generalized linear mixed-effects model with a binomial error distribution and logit link function, with herd included as a random effect. Sum contrasts (*contr.sum*) were applied to the predictors, allowing estimation of level-specific effects relative to the grand mean. Odds ratios (ORs) and corresponding 95% confidence intervals (CIs) were obtained by exponentiating the estimated regression coefficients.

All graphical outputs were generated in R v4.4.3 for Windows using the ggplot2 package [44], unless otherwise stated (GraphPad Prism v10.6.1 for Windows). The sunburst diagram was produced using the Plotly library v6.5.1 in Python [45]. Descriptive statistics were calculated in Microsoft^®^ Excel^®^ for Microsoft 365 MSO (Version 2509 Build 16.0.19231.20246) 64-bit, and regression analyses were performed in R using the lme4 package [46]. Shannon index distributions were compared between dairy and fattening herds using the Mann–Whitney U test in GraphPad Prism. Statistical significance was defined as *P*≤0.05 for all tests.

### Ethical approval

The trial was conducted in line with national and international guidelines for the care and use of animals, and approval was given by the Norwegian Food Safety Authority, Oslo, Norway (approval number 26090).

## Results

### High-quality assemblies of *P. multocida* subsp. *multocida*

Of the 370 Norwegian isolates, 365 assemblies passed the quality control thresholds and were included in downstream analyses (Table S2; Norwegian Metadata-Dataset 1). Assemblies contained an average of 2,239 CDSs, comparable to the reference genome (Table S3). One NCBI assembly was identified as *P. multocida* subsp. *septica*, based on ANI (≥98%), while four were assigned to subsp. *gallicida* based on *gatD* gene detection (Table S4). The remaining NCBI assemblies and all Norwegian assemblies were classified as subsp. *multocida* (Fig. S2; Norwegian-NCBI-Dataset 2).

### Capsular type A, LPS group 3 and ST79 as the most prevalent genotypes

**Fig. 1.**
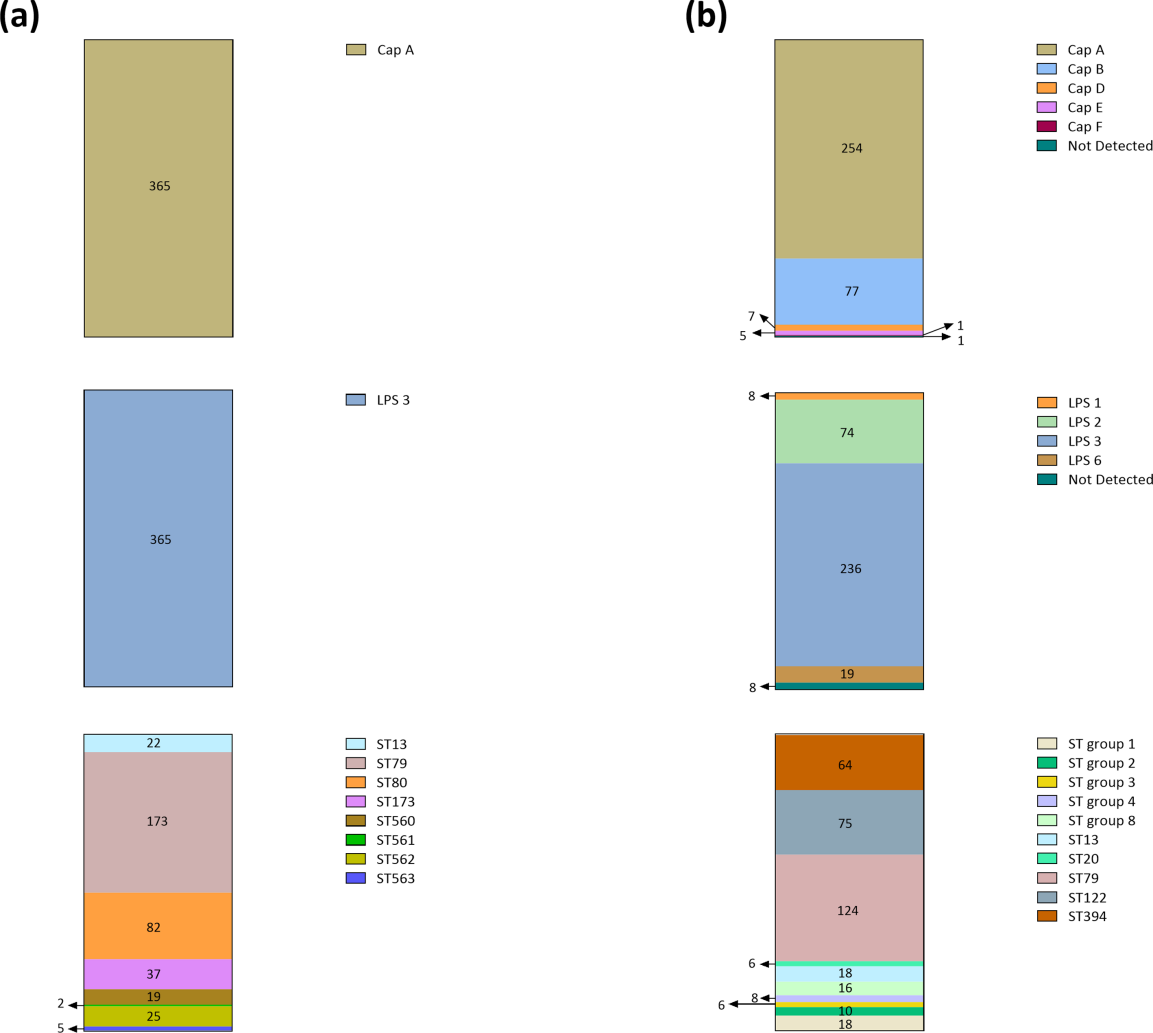
Distribution of capsular types (Cap), LPS groups and STs among bovine PM genomes. (**a**) Norwegian isolates (*n*=365). (**b**) Publicly available NCBI assemblies (*n*=345). Numbers represent the frequency of isolates assigned to each trait. Rare STs were grouped by frequency: Group 1 (single genome: ST7, ST17, ST36, ST50, ST51, ST58, ST62, ST65, ST131, ST173, ST206, ST327, ST396, ST397, ST398, ST452, ST459 and ST474), Group 2 (two genomes: ST9, ST124, ST125, ST185 and ST292), Group 3 (three genomes: ST162 and STs not detected), Group 4 (four genomes: ST132 and ST167) and Group 8 (eight genomes: ST80 and ST349). ‘Not detected’ indicates genomes for which the capsular type, LPS group or ST could not be assigned. The figure was generated using GraphPad Prism.

All Norwegian isolates were capsular type A and LPS group 3 (Fig. 1). Four known STs were identified: ST79 (*n*=173 isolates, 47.4%), ST80 (*n*=82 isolates, 22.5%) and ST13 (*n*=22 isolates, 6%), all belonging to clonal complex 13 (CC13), also ST173 (*n*=37 isolates, 10.1%), unassigned to CC. Four novel STs were identified and submitted to the RIRDC database: ST560 (*n*=19 isolates, 5.2%), ST561 (*n*=2, 0.5%), ST562 (*n*=25 isolates, 6.8%) and ST563 (*n*=5, 1.4%).

The NCBI assemblies (*n*=345) exhibited greater diversity in capsular types (A, B, D, E and F), LPS groups (1, 2, 3 and 6) and STs (34 STs; Fig. 1). Most NCBI genomes were capsular type A (*n*=254, 73.6%), LPS group 3 (*n*=236, 68.4%) and ST79 (*n*=124, 35.9%).

### Variant-based ARG and ST-specific VF patterns

**Fig. 2.**
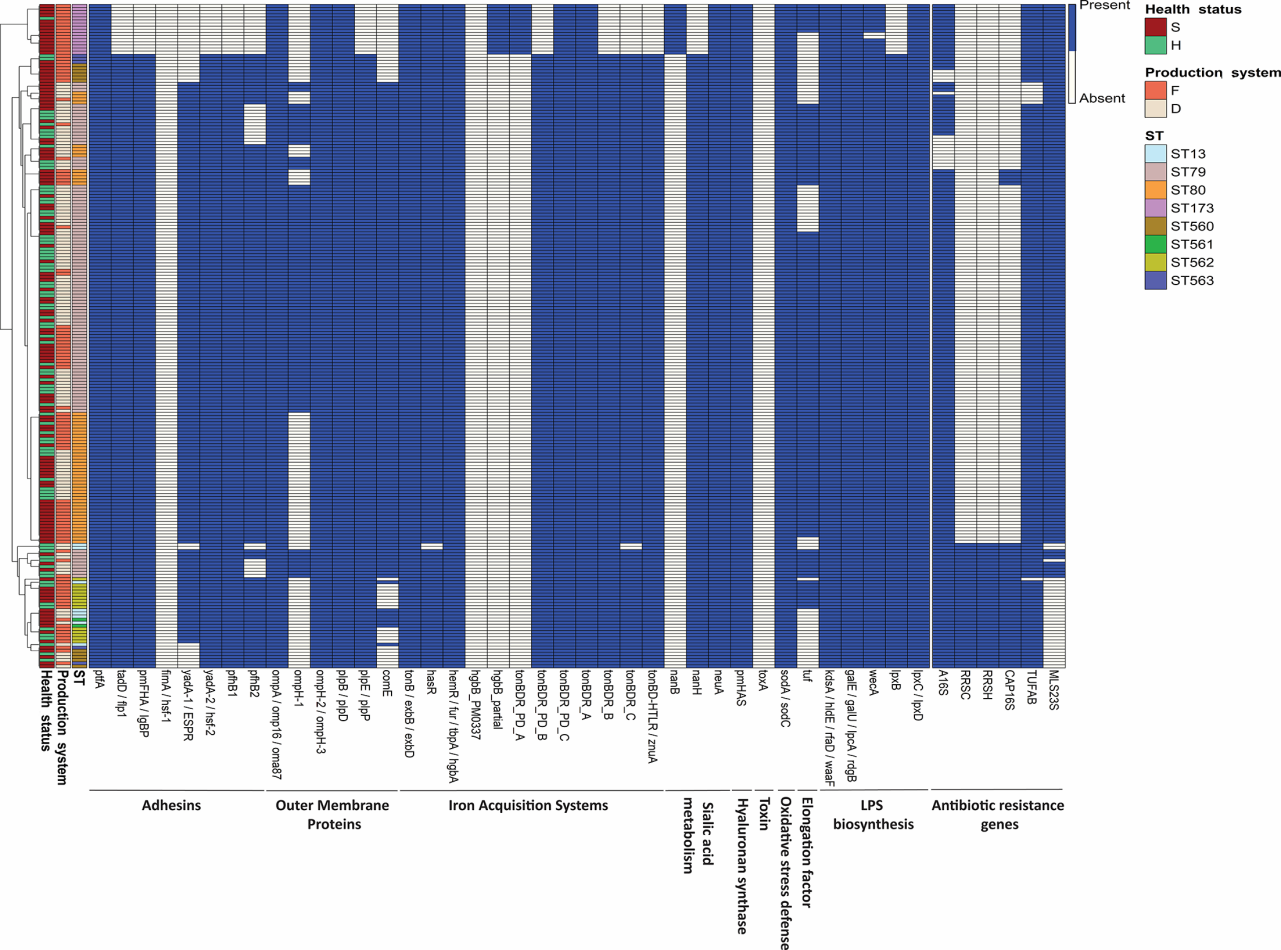
Heatmap of VFs and ARGs detected in Norwegian bovine PM isolates using tBLASTn and BLASTn, respectively. Combined labels (slashes) indicate identical presence–absence patterns of the VFs across isolates within the same functional category. Health status: S, sick (BRD); H, healthy. Production system: F, fattening; D, dairy.

No acquired ARGs were identified in Norwegian isolates. However, six point mutation-associated ARGs were detected. Mutations in the 16S rRNA gene (A16S with or without RRSC and RRSH), associated with reduced aminoglycoside binding, were present in 346 out of 365 isolates (94.8%) across all STs. Mutations in elongation factor Tu (TUFAB), linked to reduced susceptibility to elfamycins, were detected in 357 isolates (97.8%) distributed in all STs. Ribosomal binding site mutations associated with macrolide (MLS23S) and cationic antimicrobial peptide resistance (CAP16S) were identified in 303 (83%) and 80 (22%) isolates, respectively. Specifically, MLS23S was absent in ST13, ST561 and ST562, while CAP16S was absent in ST173 (Fig. 2). No plasmid-associated sequences or ICEPmu1 were detected in Norwegian isolates (Table S5).

Identified VFs were primarily linked to iron regulation and acquisition, followed by adhesion and membrane-associated proteins (Fig. 2; Table S6; Norway-Cap-LPS-ST-VF-ARG dataset). VFs were grouped (VG1–VG3) based on identical presence–absence profiles across isolates (Table S7). The *ompH1* gene and the co-occurrence of *hgbB*, *nanB* and *tonBDR plug domain-containing protein A* (*tonBDR-PD-A*) were observed to follow the clonal frames of ST79 and ST173, respectively. The *hgbB*_*nanB*_*tonBDR-PD-A* combination was significantly associated with disease (OR=4.79; 95% CI: 1.15–19.97; *P*=0.031), while the presence of *ompH1* was associated with a decreased likelihood of disease (OR=0.28, 95% CI: 0.12–0.65, *P*=0.003).

Among NCBI isolates, *ompH1* was enriched in ST79 genomes (75%) and absent from ST173 and other CC13 isolates (except for one ST13 genome). Conversely, *hgbB*, *nanB* and *tonBDR-PD-A* were present in ST173 but rare in most other STs (Tables S6 and S7; NCBI-Cap-LPS-ST-VF dataset).

### ST-based clustering and low genomic diversity among Norwegian isolates within a global context

**Fig. 3.**
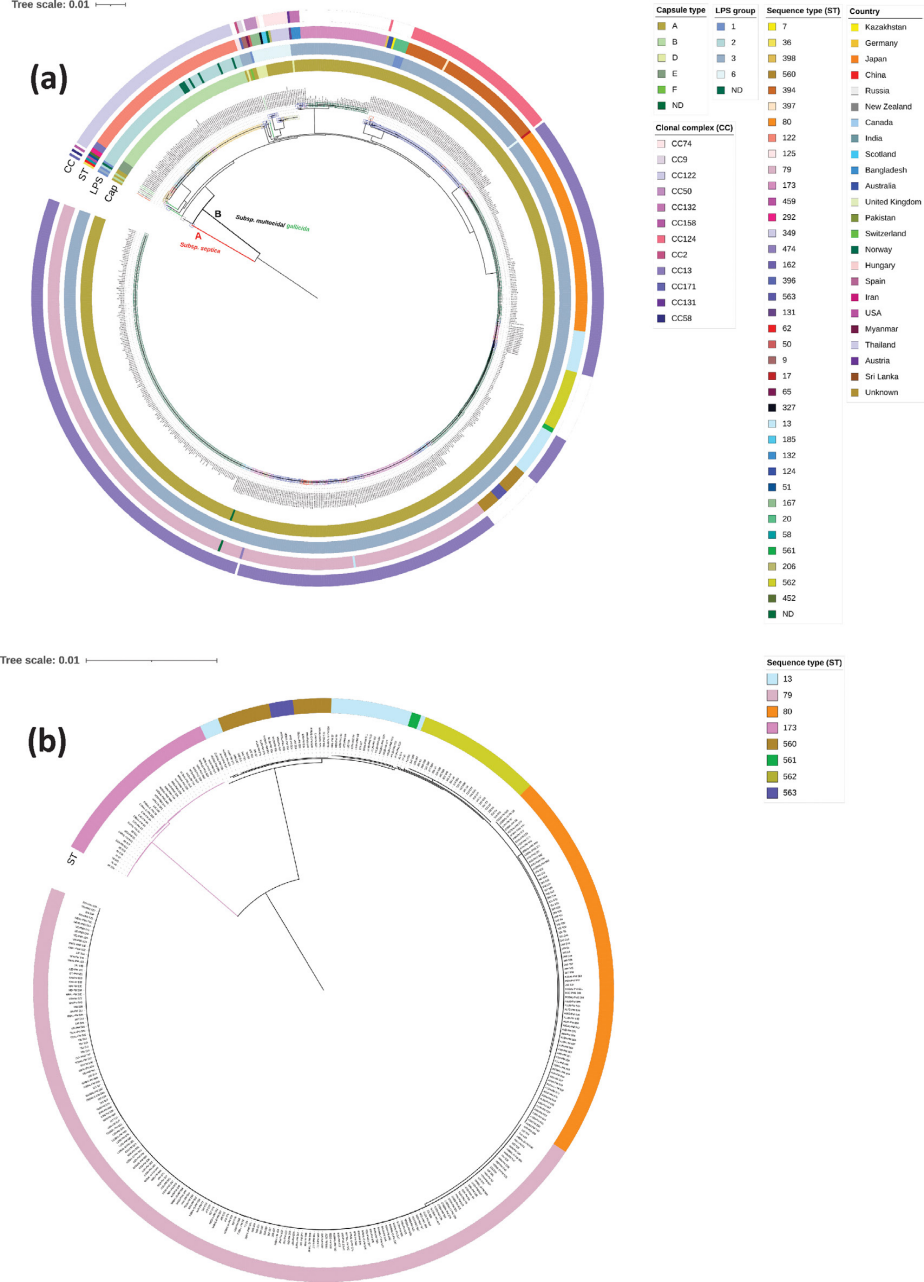
Core-genome phylogenies of PM inferred from core-gene alignments generated with Panaroo. (**a**) Phylogeny of 710 isolates, including Norwegian assemblies from this study and publicly available bovine genomes from NCBI. Annotation rings are shown from the inside out as follows: capsular type (Cap), LPS group (LPS), ST based on RIRDC MLST (ST) and clonal complex (CC). (**b**) Phylogeny of 365 Norwegian isolates. Trees were inferred using the maximum likelihood method implemented in IQ-tree, and trees were visualized by iTOL. Shared tree links are available at https://itol.embl.de/export/12839239142111821746706521 and https://itol.embl.de/export/891121570464821767098310.

The phylogeny of the Norwegian-NCBI-Dataset 2 (*n*=710) revealed two major clades (Fig. 3a). Clade A (in red) consisted of a single, highly divergent genome of PM subspecies *septica* (capsular type A, LPS type 1 and ST36), consistent with its assignment based on ANI analysis. Clade B included the remaining genomes, primarily subspecies *multocida* (black) and four subspecies *gallicida* (green). This clade formed multiple nested subclades, dominated by capsular type A, LPS type 3 and ST79 (41.8%). Notably, isolates clustered by ST rather than geographic origin, except for ST13, which appeared polyphyletic and heterogeneous.

All Norwegian isolates (Norwegian Metadata-Dataset 1; *n*=365) clustered within Clade B and were distributed between two subclades with limited intra-clade diversity (Fig. 3a, b). One subclade consisted exclusively of ST173 assemblies, which clustered together with an NCBI ST173 genome from the USA and formed a sister clade to four ST132 isolates from Australia. The second subclade included the remaining Norwegian STs, which nested within the diversity of the global NCBI isolates of ST13, ST79 and ST80. The novel Norwegian STs (ST560, ST561, ST562 and ST563) were interspersed among these established STs, indicating shared ancestry.

### The MST divided the Norwegian PM into three ST clusters

The initial wgMLST schema was generated from 365 Norwegian assemblies comprising 2,753 loci. Allele calling identified 3,944 novel alleles, expanding the schema to 6,697 alleles. Two paralogous loci were detected and removed. Core genome loci, defined as those present in ≥95% of genomes, yielded a cgMLST schema consisting of 1,620 loci.

**Fig. 4.**
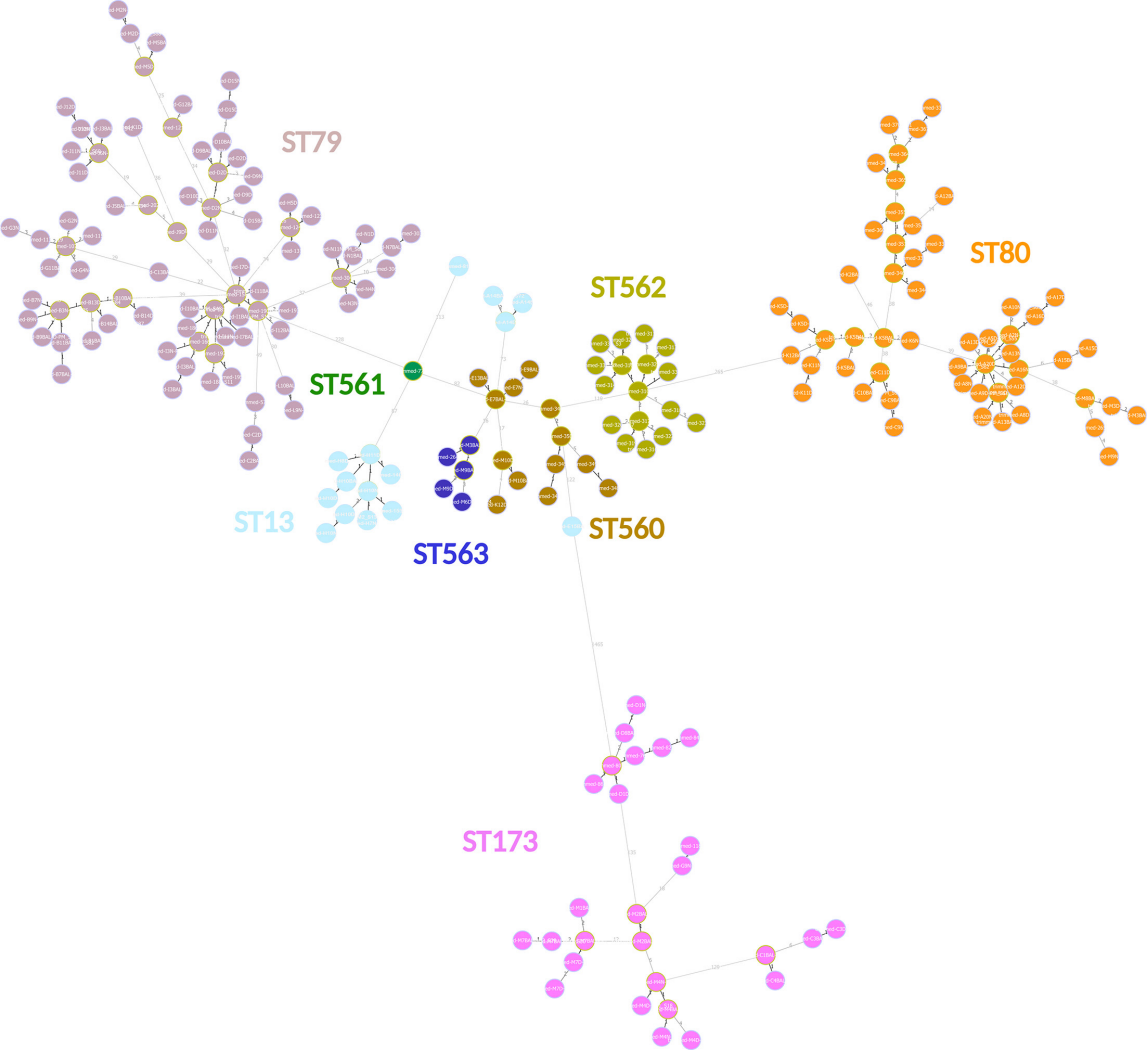
MST of Norwegian PM isolates based on 216 non-redundant cgMLST95 profiles, generated using the goeBURST algorithm in Phyloviz v.2. STs are indicated by colours and labels. An MST including both Norwegian and NCBI isolates is provided in Fig. S3.

A total of 149 isolates sharing identical cgMLST profiles with other isolates were excluded from the MST, retaining a single representative per profile, which resulted in 216 non-redundant isolates in the final MST (Fig. 4). The MST indicated three main clusters corresponding to ST79, ST80 and ST173. These clusters were connected by ‘bridging clusters’ of ST13, ST560, ST561, ST562 and ST563. The smallest allelic difference between isolates of various STs was 16 alleles (mean: 25.5±6.7), observed between ST560 and ST563, whereas the largest difference was 1,554 alleles (mean: 1545.2±8.9) between ST79 and ST173 (Table S8). Allelic differences within each ST varied widely, from 0 (identical isolates) to a maximum average of 87.9 (sd±62.9) in ST173, 67.2 (sd±60.9) in ST13 and 38.1 (sd±13.9) in ST79. Notably, ST13 isolates were dispersed across the MST, separated by 108 to 182 alleles (mean: 121.8±15.6; Table S9).

### Inter- and intra-herd diversity – a more diverse population of PM was observed in fattening herds

**Fig. 5.**
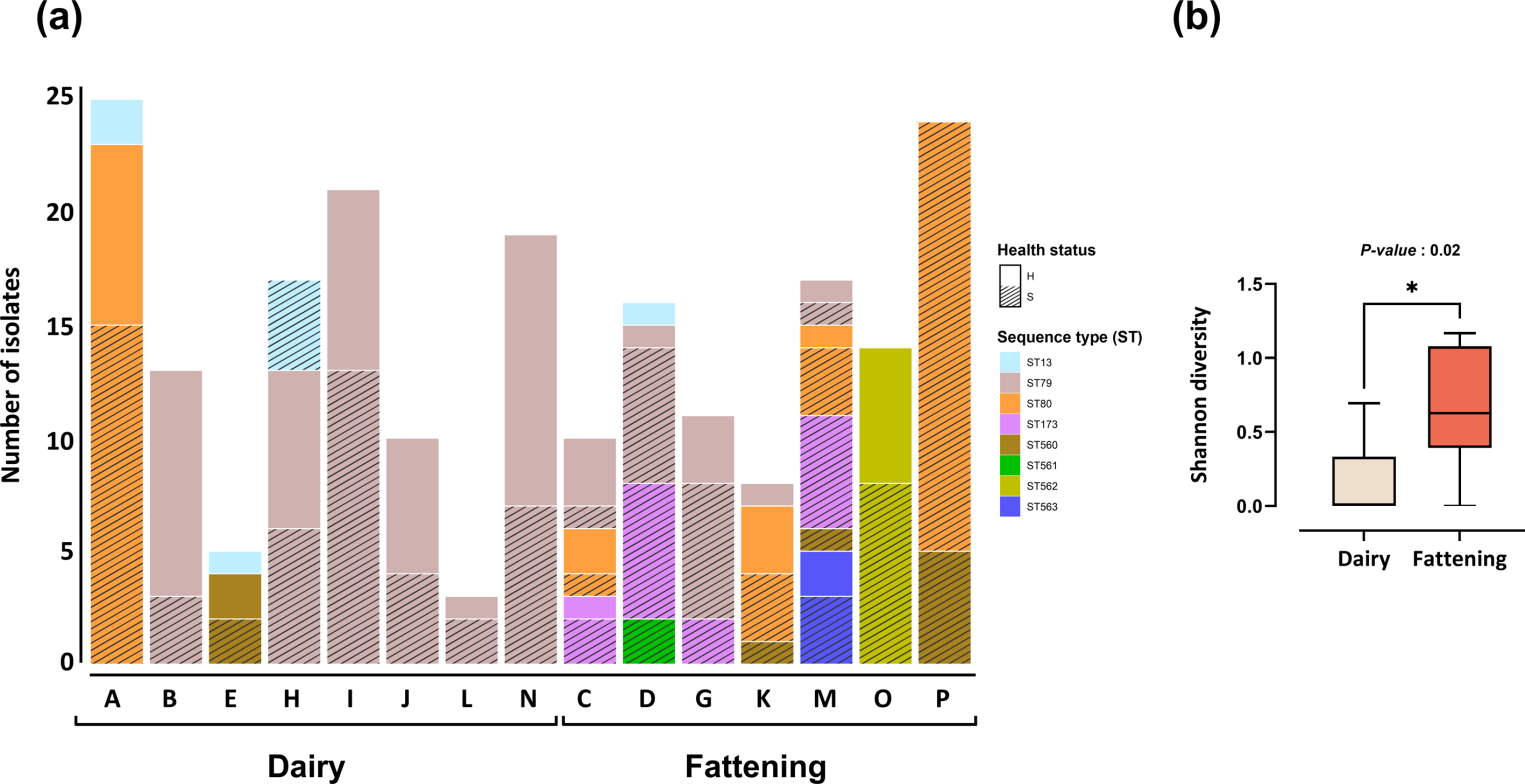
(a) Distribution of circulating STs in bovine PM isolates from 16 Norwegian herds across two production systems, dairy and fattening herds. Herds are labelled as letters A to P; herd F (dairy) was excluded as no PM was detected. Animal health status is indicated as S (sick; BRD) and H (healthy). In Ånestad *et al.*’s study [24], herd labels were reassigned chronologically by production system across two publications (dairy first; fattening, unpublished). In the present study, the original herd labels were retained to maintain consistency with sequence identifiers (see Table S1 for differences between labelling schemes). (**b) **Box plot (made in GraphPad Prism) showing differences in Shannon diversity index between production systems. *P-*value is based on the Mann–Whitney U test.

Norwegian herds harboured between one and five STs simultaneously (Fig. 5a; Table 3), with fattening herds exhibiting significantly higher diversity than dairy herds (Shannon’s H index 1.7 vs. 0.9; Fig. 5b). ST173, ST561, ST562 and ST563 were detected exclusively in fattening herds (Fig. 5a).

**Table 3.** Circulating STs of PM isolates from healthy and BRD-affected calves across herd production systems. Values represent the ratio of isolates to calves from which the isolates were recovered. The original dataset of 365 isolates was reduced to 213 by collapsing isolates with ≤5 allelic differences based on a goeBURST allelic differences, and identical virulence and AMR profiles into a single representative. This avoided the overrepresentation of genetically identical isolates from the same animal

ST	Total	Healthy (*n*=82/58)		BRD cases (*n*=131/91)	
		Dairy (*n*=84/40)	Fattening (*n*=38/18)	Dairy (*n*=115/38)	Fattening (*n*=128/53)
**ST13**	8/7	3/2	1/1	4/4	0
**ST79**	102/70	44/30	9/7	35/22	14/11
**ST80**	55/40	8/6	6/6	15/10	26/18
**ST173**	16/15	0	1/1	0	15/14
**ST560**	11/11	2/2	0	2/2	7/7
**ST561**	2/2	0	0	0	2/2
**ST562**	14/9	0	6/3	0	8/6
**ST563**	5/3	0	2/1	0	3/2

**Fig. 6.**
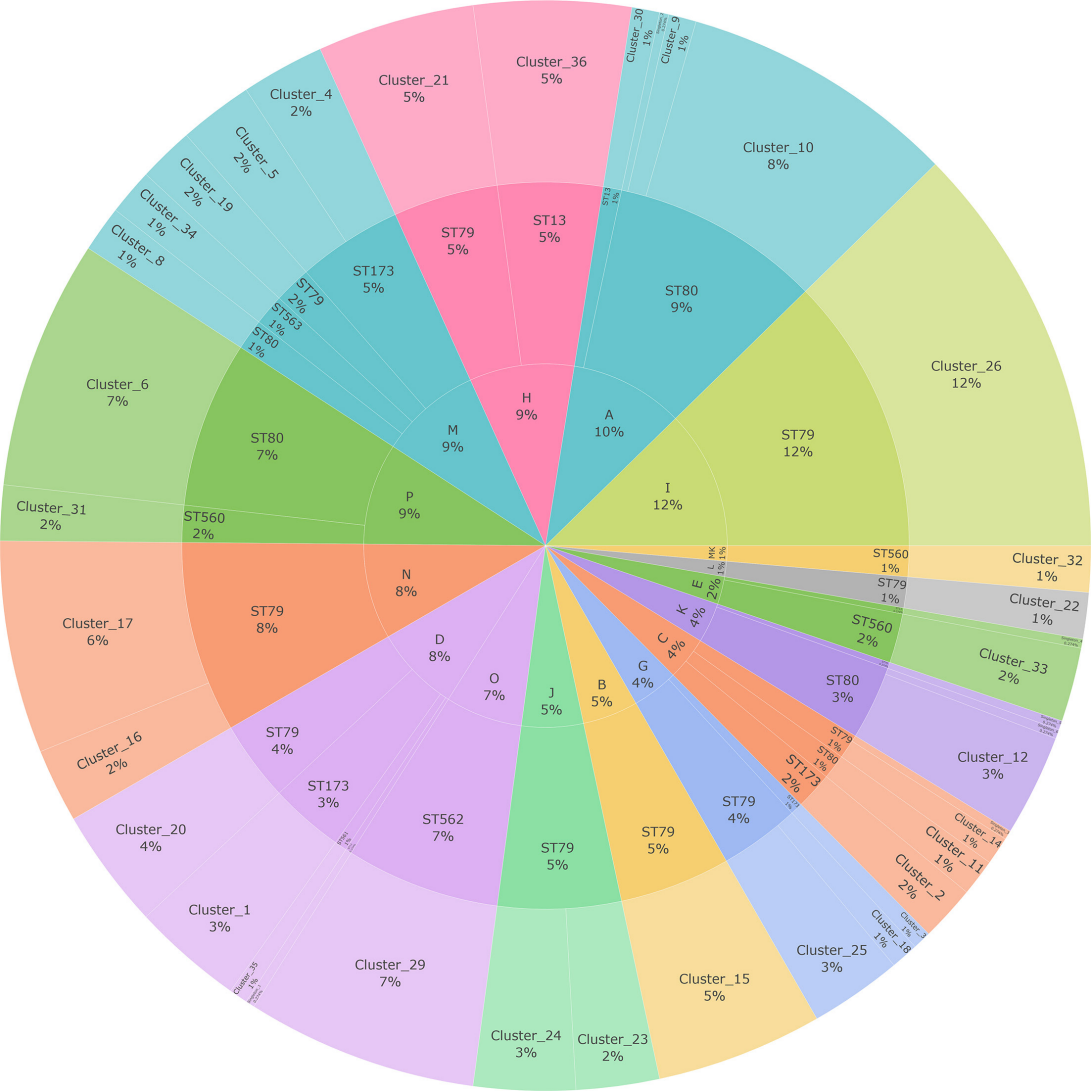
Sunburst diagram showing hierarchical relationships between herds (inner ring), STs (middle ring) and ReporTree Clusters (outer ring). Segment widths are proportional to the number of *P. multocida* isolates, with colours distinguishing herds, and labels identifying STs and clusters. The diagram illustrates genetic diversity within herds and the sharing of STs and clusters across herds. An interactive version of this figure is provided here: https://akllarena.github.io/aahmadi26-sunburst-figure/.

ReporTree grouped 365 Norwegian isolates using hierarchical clustering with a threshold of ≤5 allelic differences. Of these, 359 isolates formed 32 clusters (2 to 45 isolates per cluster), while 6 isolates remained as singletons. ReporTree Clusters followed the ST clonal frame but increased resolution beyond ST, subdividing ST79, ST80, ST173, ST560 and ST13 into 13, 6, 5, 3 and 2 ReporTree Clusters, respectively (Fig. 6). Only ReporTree Cluster 32 comprised isolates from two different herds, with five ST560 isolates collected from two calves in fattening herds M and K (Fig. 6).

Most isolates from the same herd and ST clustered together and also shared similar VF and AMR profiles, irrespective of clinical status or sampling site. In six mono-ST herds (B, I, J, L, N and O; five of which were dairy), isolates formed herd-specific ReporTree Clusters. Herd B included 18 clonal isolates from 11 calves (Cluster 15), of which 13 isolates (8 calves) also shared identical VF and AMR profiles. Likewise, all isolates from herd I (12 calves, Cluster 26) were clonal and identical in VF–AMR content. Herds N and J each harboured two ST79 clusters (Fig. 6).

Most multi-ST herds (herds C, D, H, K and P; predominantly fattening herds) had a one-to-one correspondence between the number of ReporTree Clusters and STs, after excluding singletons. In contrast, herds A, G and M exhibited greater within-ST diversity, with three, three and six ReporTree Clusters across two, two and five STs, respectively. In herd A, 33 of 36 isolates belonged to ST80, distributed into ReporTree Clusters 9 (*n*=3) and 10 (*n*=30). Similarly, in herd G, 13 of 15 isolates were ST79, forming ReporTree Clusters 18 (*n*=3) and 25 (*n*=10). Calves in herd M carried isolates of five STs (ST79, ST80, ST560, ST563 and ST173), with ST173 further divided into ReporTree Clusters 4 and 5.

Singletons included two isolates from herd K (ST79 and ST80), and one isolate each from herds A (ST80), C (ST79), D (ST13) and E (ST13). Three singletons differed by 41 to 49 alleles from other isolates in the collection, while the remaining singletons showed even greater allelic divergence. Notably, a singleton BAL isolate in herd A (ST80) differed by only 13 alleles from ReporTree Cluster 6 (ST80, *n*=27 isolates) in fattening herd P, whereas the corresponding N and D isolates from the same calf clustered with the remaining ST80 isolates in herd A. Consistently, the ST79 singleton in herd C deviated by 21 alleles from the large ST79 ReporTree Cluster 26 identified in dairy herd I.

### Intra-individual variations (N, D and BAL)

Of the 149 calves included in this study, 11 had PMs assigned to more than 1 ReporTree Cluster (Table 4). In ten calves, isolates were distributed across different ReporTree Clusters within the same herd. The remaining calf, 12K, harboured isolates belonging to two ReporTree Clusters: one specific to herd K (ReporTree Cluster 12) and another shared between herds K and M (ReporTree Cluster 32).

**Table 4.** Individuals for whom multiple PM isolates were assigned to more than one ReporTree Cluster. (A) Individuals with multiple cluster assignments occurred within the same herd. (B) The individual (12K) with isolates assigned to two clusters spanning different herds, including Cluster_32, which contained isolates from herds M and K

Individual	Herd	Clusters appearing in	Notes
**2D**	D	Cluster_1, Cluster_20	A
**7D**	D	Cluster_1, Cluster_35	A
**2M**	M	Cluster_5, Cluster_19	A
**3M**	M	Cluster_8, Cluster_34	A
**7M**	M	Cluster_4, Cluster_8	A
**9M**	M	Cluster_8, Cluster_34	A
**4N**	N	Cluster_16, Cluster_17	A
**8N**	N	Cluster_16, Cluster_17	A
**12** N	N	Cluster_16, Cluster_17	A
**2P**	P	Cluster_6, Cluster_31	A
**12K**	K	Cluster_12, Cluster_32	B

To assess the diagnostic comparability of different sampling methods, we evaluated the clonality of isolates obtained from all three respiratory tract sites (N-D-BAL) within the same animal using the Triple-site-isolate-Dataset. A stringent criterion for clonality was applied (≤5 goeBURST allelic differences with identical VF and AMR profiles), resulting in Dataset 3 (*n*=213 isolates). Including VF and AMR profiles enabled evaluation of whether the sampling method captured traits relevant to clinical outcomes. Isolates recovered from all three sites were clonal in 50% of the cases, while N-D pair-isolates were more often clonal than N-BAL or D-BAL pairs (Table 5).

**Table 5.** Comparison of PM isolates (Triple-site-isolate-Dataset) recovered from three respiratory tract sites per animal: N, D and BAL. Comparisons were based on (i) ST diversity and associated VF and AMR profiles and (ii) cgMLST95 relatedness (≤5 allele differences), combined with VF and AMR profiles. Some clonal isolates (≤5 alleles) differed in VF or AMR profiles

	% of calves with similar N-D	% of calves with similar N-BAL	% of calves with similar D-BAL	% of calves with similar N-D-BAL
**Identical ST+similar AMR/VF**	75.9	62.1	67.2	53.4
**Clonal isolates+similar AMR/VF**	74.1	60.3	63.8	50

### Associations with BRD

The herd production system was significantly associated with clinical signs of BRD. Calves from dairy herds had significantly lower odds of showing clinical signs (OR=0.57; 95% CI: 0.36–0.89; *P*=0.014), while calves from fattening herds had significantly higher odds (OR=1.77; 95% CI: 1.12–2.79; *P*=0.014).

A significant association was observed between specific STs and clinical status. ST173 was strongly associated with disease, as calves carrying an ST173 isolate had higher odds of being clinically affected than those with other STs (OR=6.35; 95% CI: 1.11–36.29; *P*=0.038). In contrast, ST79 was associated with significantly lower odds of clinical disease (OR=0.35; 95% CI: 0.16–0.76; *P*=0.0075). No other STs were significantly associated with clinical status.

**Fig. 7.**
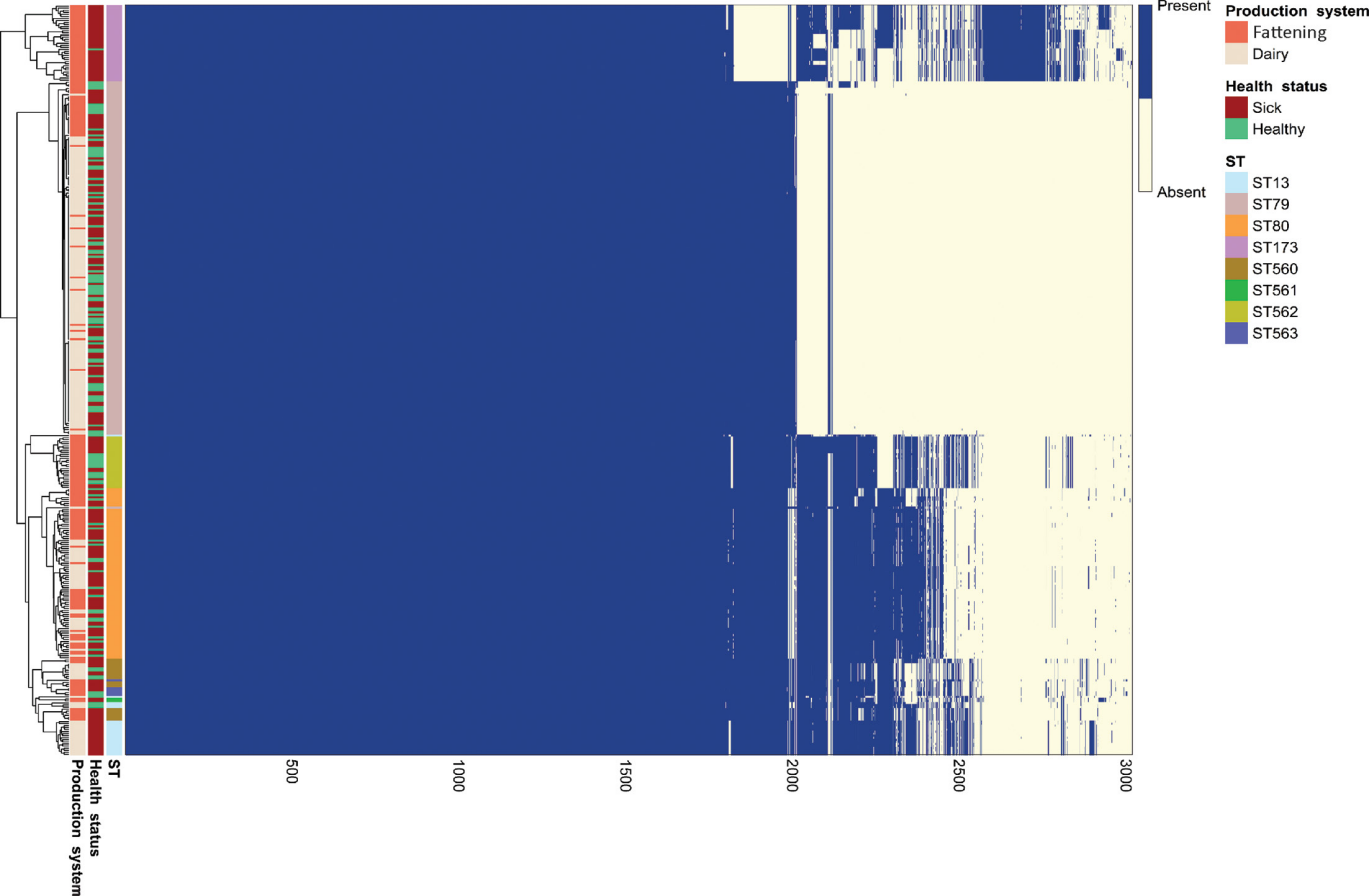
Pangenome structure of Norwegian PM genomes. Pangenome analysis was performed using Panaroo, and gene presence–absence heatmaps depicting the distribution of core and accessory genes were generated in R. The heatmap shown includes Norwegian isolates only; the corresponding heatmap for combined Norwegian and NCBI bovine assemblies (*n*=710) is provided in Fig. S4.

**Fig. 8.**
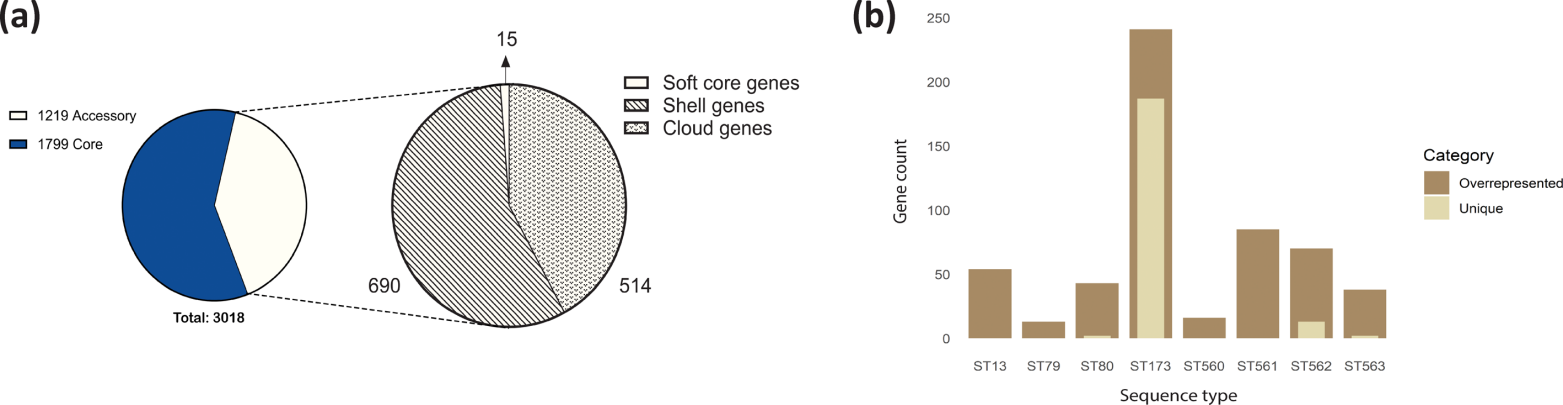
(**a**) Proportion of genes across the core (present in 99–100% of isolates) and accessory genomes of Norwegian PM isolates. The accessory genome was subdivided into soft core (95–99%), shell (15–95%) and cloud (0–15%) genes based on Panaroo. **(b) **Overrepresented and unique genes identified among STs using Scoary.

The accessory-genome clusters largely followed the core-genome clonal frame, indicating that variation in accessory-gene content is primarily introduced through mobile genetic elements at the lineage level rather than through homologous recombination. ST173 exhibited a distinct accessory genome profile (Fig. 7), characterized by a unique set of genes (*n*=187; Fig. 8) and the absence of another set (*n*=158). GWAS indicated that ST173 harboured the largest number of overrepresented and unique genes compared to all other STs (Scoary_ST173 dataset). The GWAS analysis for health status did not find any genes associated with BRD.

**Fig. 9.**
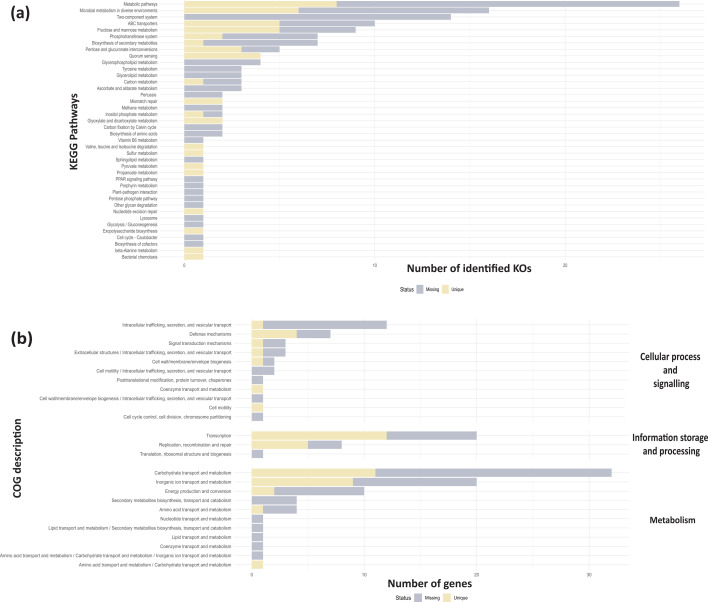
Functional annotation of unique (*n*=187) and missing (*n*=158) genes in PM ST173. (a) KEGG pathway classification showing the distribution of genes across metabolic pathways based on KEGG Orthology (KO) annotations. (**b**) COG functional classification showing the distribution of genes across the COG categories.

KO identifiers were assigned to 63 (34%) of the ST173-unique protein sequences and 85 (54%) of the missing genes. Several pathways were linked to both sets, with metabolic pathways being the most enriched in both unique and missing genes (Fig. 9a). The two-component system pathway was among the top-enriched pathways for missing genes, while quorum sensing was prominent among unique genes. Functionally, KO identifiers in both gene sets were predominantly associated with metabolism (24 KOs in unique genes; 52 KOs in missing genes), followed by genetic information processing (17 unique; 12 missing) and cellular process and signalling (10 unique; 24 missing) (Fig. 9b).

## Discussion

This study characterizes the population structure, within-host diversity and transmission dynamics of PM in Norwegian calves. As a major contributor to BRD, understanding the epidemiology of PM is critical for improving disease control strategies.

### Population structure of PM

All isolates in our study were *P. multocida* spp. *multocida*, capsular type A and LPS type 3, demonstrating the dominance of this serotype in Norwegian cattle herds with enzootic pneumonia. Globally, capsular type A with LPS type 3 is common among bovine PM isolates, although other capsular types (B, D, E and F), LPS serogroups (1, 2 and 6) and subspecies (*gallicida/septica*) are also reported [7,47]. This serotype has been identified across multiple hosts and disease manifestations, including avian fowl cholera, progressive atrophic rhinitis and pneumonic pasteurellosis in pigs [7,21].

ST79 was the most prevalent ST, followed by ST80, ST173, ST562 and ST13. Members of CC13, including ST79, ST80 and ST13, are frequently associated with BRD, with ST79 being the most commonly reported bovine lineage globally [7,18, 33, 48]. Its rare detection in other hosts [18,19, 49, 50] suggests cattle adaptation, potentially driven by lineage-specific genetic features enhancing fitness in this host species [7]. *OmpH-1* was enriched in ST79 but absent in other CC13 isolates, suggesting a potential role in bovine colonization. While OMPs have been proposed as vaccine targets in PM [51], and the high prevalence of *OmpH-1* in bovine ST79 isolates makes it a candidate for future vaccine development, this genomic association requires functional and immunological validation.

ST13 has been isolated from various host species [19,21, 33, 50]. The *tbpA* gene of ST13 has previously been proposed as an epidemiological marker for ruminant hosts, as it is highly prevalent among ruminant PM isolates but rarely found in avian/porcine hosts [7,18, 52,55]. Constantly, all Norwegian ST13 isolates and 99.7% of NCBI bovine isolates carried *tbpA*, supporting that PM with capsular type A, LPS type 3 and ST13 harbouring *tbpA* may be adapted to bovine hosts.

The relatively high prevalence of ST173 among Norwegian calves aligns with recent findings from Sweden, suggesting regional enrichment of this lineage in Nordic cattle populations. Comparable geographic structuring has previously been reported for ST394 in Australia [47] and for other bacterial species, such as the overrepresentation of ST45 and ST677 among zoonotic *Campylobacter jejuni* in Nordic countries [56,57].

A limited set of point mutations potentially associated with reduced susceptibility to aminoglycosides, elfamycins, macrolides and cationic antimicrobial peptides was widely detected. Their broad distribution across STs suggests that they represent intrinsic genomic features rather than recently acquired resistance. These findings align with the phenotypic resistance reported by Ånestad *et al.* [24], which showed streptomycin resistance in the majority of Norwegian PM isolates. The absence of acquired resistance determinants reflects the favourable AMR situation in Norway, as also documented by the NORM-VET programme [58]. This supports the interpretation that AMR in this population is primarily mutation-driven.

Phylogenetic analysis showed that the Norwegian isolates clustered into multiple clades alongside global isolates, suggesting multiple introductions rather than a single recent ancestor. Some Norwegian isolates clustered with European isolates (UK, Switzerland and Germany), potentially reflecting historical livestock trade or shared reservoirs. Although the import of live cattle into Norway is very limited and unlikely to represent a major introduction route [59], imports of small ruminants and camelids (llamas, alpacas and camels) do occur and are considered a potential risk for introducing contagious diseases into cattle populations [59,60]. Alternatively, the observed phylogenetic patterns may reflect the presence of globally distributed bovine-adapted lineages, as supported by the clustering of Norwegian isolates with strains from Asia and the USA. Such weak geographical structuring is consistent with pathogens primarily selected by host adaptation rather than geographic constraints [61,64].

### Inter-herd and intra-herd variation

Clear genetic differences were observed between herd production systems, with herd-specific population structures and varying numbers of co-circulating STs. Diversity ranged from one to five STs per herd and was consistently higher in fattening herds than in dairy herds. Several STs, including three novel types, were detected exclusively in fattening systems. This suggests that fattening herds may facilitate the emergence, persistence and amplification of lineages due to management practices, such as group mixing, frequent animal turnover and recruitment from multiple sources [19,65]. In contrast, dairy herds typically have a more closed structure, with self-recruitment of calves and lower replacement rates, which limit diversity and favour the dominance of single PM lineages within herds.

Despite the presence of shared STs across herds, cgMLST analysis revealed largely herd-specific PM populations, indicating limited inter-herd transmission. The detection of a single shared cluster (ReporTree Cluster 32, five isolates from two calves) between two fattening herds likely reflects either recent inter-herd transmission or exposure to a common external source. As the herds were geographically dispersed and unlikely to share veterinarians, transport vehicles or animal sources, no clear epidemiological link could be identified.

These findings suggest that once a lineage is introduced, it tends to expand clonally within individual herds with minimal inter-herd spread. However, as this study focused on only 15 herds with persistent BRD, transmission dynamics may differ in herds experiencing acute outbreaks. Moreover, Norwegian herds are generally smaller than those in many other countries, which may influence population structure and transmission dynamics. While these factors strengthen the internal validity of the findings, caution should be taken when generalizing to herds with different management practices, larger herd sizes or acute outbreak situations.

### Intra-animal variation and diagnostic accuracy of N, D and BAL samples

Most calves carried a single dominant PM clone. However, 11 calves harboured multiple clones, indicating within-host heterogeneity. For ten of these calves, the clones belonged to herd-specific clusters, suggesting mixed infections originating from clones within herds. This is consistent with calves being exposed to and may transiently carry several co-circulating lineages in fattening herds with higher PM diversity. A notable exception was calf 12K, whose isolates clustered both within herd K-specific Cluster 12 and the mixed herd M–K Cluster 32, providing evidence of either inter-herd transmission or cross-contamination.

Whether upper respiratory tract isolates accurately represent pathogenic lineages in the lower respiratory tract, which are the primary targets for antimicrobial treatment, remains debated [66,67]. To address this, we applied a stringent definition of clonality to assess whether isolates were identical with respect to disease-relevant traits. Agreement between N and D swabs was highest (74.1%) but was lower for comparisons involving BAL isolates and below that reported in a comparable study from Sweden (75%) [67]. Only half of the calves carried clonal isolates across all three sampling sites (N–D–BAL). These findings suggest that lineages colonizing the lower respiratory tract may differ from those present in the upper airways. This variation could be due to niche specialization or within-host microevolution and horizontal gene transfer, leading to accessory genome turnover. Additionally, since draft genomes were used, technical factors like assembly fragmentation may have reduced the detection of certain genes, potentially underestimating the concordance between sites. Nevertheless, the low agreement between BAL and upper respiratory samples indicates that relying solely on upper airway sampling may fail to detect virulent or resistant PM clones in the lower airways. This is critical for outbreak investigations and targeted antimicrobial therapy.

### Genotypic predictors and clinical outcomes

Herd production system emerged as a key predictor of disease; calves from fattening herds showed significantly higher odds of respiratory tract disease than those from dairy herds. Despite the limited sample size (*n*=15 herds) and the possibility that clinical examination does not fully capture underlying infection or disease processes, the observed pattern aligns well with established risk factors, including higher stocking densities, multi-source sourcing, weaning stress and transport, all of which facilitate pathogen transmission and impair immunity [68,70].

A strong association was observed between ST173 and clinical disease (over six-fold increased odds). ST173 was also more frequently isolated from BAL samples, suggesting a possible tropism for the lower respiratory tract. Its consistent presence in BRD cases across studies [67] further supports its potential importance in BRD pathogenesis, particularly in fattening herds. Although GWAS did not identify genes significantly associated with BRD, ST173 formed a genetically distinct cluster with a characteristic pangenome profile, including 187 uniquely present and 158 absent genes. ST173 lacked several common VFs but carried a specialized repertoire for host colonization and iron acquisition, including *hgbB*, *nanB* and *tonBDR-PD-A.* The sialidase encoded by *nanB* facilitates sialic acid utilization and mucosal colonization [20], while the concurrent presence of *hgbA*, *hgbB* and the *tonBDR-PD-A* demonstrates an expanded capacity for iron and haem acquisition under host-imposed iron limitation [71,72]. Functional analysis of genes uniquely present and absent in ST173 revealed metabolic reconfiguration consistent with niche-specific adaptation [73,74]. Notably, the loss of genes linked to two-component regulatory systems indicates reduced environmental sensing [75], whereas retention of quorum-sensing genes suggests a reliance on population-level coordination, potentially facilitating biofilm formation or evasion of host defences [76,77]. Collectively, these features suggest that ST173 represents a lineage undergoing genomic reconfiguration associated with increased pathogenic potential in the bovine respiratory tract. Functional studies are required to confirm their contribution to disease severity.

In contrast, ST79 was associated with a significantly lower likelihood of signs of respiratory disease. ST79 is the most prevalent ST reported from both healthy and BRD cases globally [19,21, 33, 47], being both a commensal and an opportunistic pathogen. Its neutral or potentially protective nature may be due to reduced virulence, competitive interactions within the microbiome or ecological factors favouring commensal over pathogenic behaviour. Comparisons with the NCBI isolates were limited by the lack of clinical metadata, constraining full assessment of genotype-disease associations, as noted previously [78]. This underscores the importance of integrating clinical data with genomic analyses to better understand the relationship between bacterial genotype and disease outcome.

The findings have practical implications for BRD control. In addition to host and environmental factors, the bacterial lineage-specific characteristics clearly influence disease risk. This highlights the importance of biosecurity and herd measurement to prevent the introduction of pathogenic lineages like ST173. Contact with animals from other herds should be limited, and fomites should not be transferred between herds. Within herds, practices like maintaining small, age-matched groups, avoiding commingling and unnecessary animal movements and using separate equipment for different groups can reduce and prevent transmission of more pathogenic strains. Moreover, for vaccine development, targeting virulent lineages rather than common commensal strains may improve vaccine efficacy.

### Limitations

As a cross-sectional study, these results represent a snapshot in time, and both prevalence and genomic characteristics of circulating PM lineages may vary over time. However, inclusion of both healthy and diseased animals across multiple herds and production systems strengthens the relevance of our findings. Nevertheless, our results may not fully capture the strain diversity present in acute outbreaks, other regions or production environments.

## Conclusions

This study provides new insights into the genetic diversity of bovine PM at multiple levels: globally, between herds in Norway, within herds and within individual animals. The overall genomic diversity of isolates circulating in Norwegian cattle was low, with all categorized as capsular type A, LPS type 3 and subspecies *multocida.* Despite this apparent homogeneity, certain STs were associated with clinical statuses and herd production systems. ST173 was strongly linked to calves showing signs of respiratory tract disease, particularly in fattening herds, and carried some unique VFs (*nanB*, *hgbB* and *tonBDR-PD-A*) that may enhance its ability to colonize the lower respiratory tract in cattle and cause disease. In contrast, ST79 was the most prevalent ST in both healthy and diseased calves, supporting its role as both a commensal and opportunistic pathogen. The findings underscore the importance of biosecurity measurements to limit the introduction and spread of pathogenic strains between and within herds. Further studies are needed to investigate the functional roles of identified VFs and longitudinal transmission dynamics and experimentally confirm their clinical impact on respiratory tract disease and the cattle production system.

## Supplementary material

10.1099/mgen.0.001735 Supplementary Material 1.

10.1099/mgen.0.001735 Supplementary Material 2.

## Abbreviations

AMR: antimicrobial resistance
ANI: average nucleotide identity
ARG: antimicrobial resistance gene
AST: antimicrobial susceptibility testing
BAL: non-endoscopic bronchoalveolar lavage
BRD: bovine respiratory disease
Cap: capsule
CC13: clonal complex 13
cgMLST: core genome MLST
CI: confidence interval
COG: Clusters of Orthologous Groups
D: guarded nasopharyngeal swab
eggNOG: Evolutionary Genealogy of Genes: Non-supervised Orthologous Groups
GWAS: genome-wide association study
ICE: integrative and conjugative elements
iTOL: Interactive Tree of Life
KEGG: Kyoto Encyclopedia of Genes and Genomes
KO: KEGG Orthology
LPS: lipopolysaccharide
MLST: multi-locus sequence typing
MST: minimum spanning tree
N: nasal swab
NCBI: National Center of Biotechnology Information
OMP: outer membrane proteins
OR: odds ratio
PM: *Pasteurella multocida*
RIRDC: Rural Industries Research and Development Corporation
ST: sequence type
VFs: virulence factors
wgMLST: whole-genome MLST
