# Supplementary material for: Genomic diversity and virulence of Pasteurella multocida in Norwegian calves
Source: Microb Genom. 2026 Jun 8;12(6):001735. doi: 10.1099/mgen.0.001735 (PMC13245661; doi:10.1099/mgen.0.001735)
Supplement: Supplementary Material 2. [file mgen-12-01735-s002.pdf]

Material and Methods

Sample collection

In a cross-sectional study, Ånestad et al. (2026) (1) investigated calves in Norwegian dairy and fattening herds with enzootic bovine respiratory disease (BRD). 16 herds across middle and southern Norway were selected based on herd size, housing system, and documented BRD occurrence, and were visited once between 2021 and 2022. In total, 219 calves were sampled.

Calves older than 10 days, both healthy and diseased, were included unless they had received antimicrobial treatment or vaccination within 14 days prior to sampling or showed severe dyspnea.

Data were collected from national registries, farmer interviews, and on-site observations. All calves underwent a standardized clinical examination performed by an experienced veterinarian using a modified scoring system (2) to classify them as healthy or diseased.

Biological samples—including nasal swabs, guarded nasopharyngeal swabs, bronchoalveolar lavage (BAL) fluid, and blood—were collected under sedation following strict protocols and stored appropriately for laboratory analysis. Bacterial cultures from respiratory samples were performed within 24 hours, and species identification was confirmed using MALDI-TOF mass spectrometry. From the sampled population, *P. multocida* was isolated from 149 calves originating from 15 herds, and only these were included in the present study.

Antimicrobial susceptibility testing was conducted by Ånestad et al. (2026) (1) on *P. multocida* isolates using the disc diffusion method. The antimicrobial panel included penicillin, amoxicillin-clavulanic acid, trimethoprim-sulfamethoxazole, tetracycline, enrofloxacin, florfenicol, and streptomycin.

Herd labelling

Herd labels were reassigned chronologically by production system in Ånestad et al. (1). However, to maintain consistency with sequence identifiers and isolate metadata used throughout the present study, the original herd labels are retained. Table S1 provides a cross-reference between the two labelling schemes.

Table S1. Correspondence between original herd labels used in the present study and herd labels reassigned in Ånestad et al. (1). Original labels were retained in this study to ensure consistency with sequence identifiers and isolate metadata.

Dairy herds		Fattening herds	
**This study**	**Ånestad et al.**	**This study**	**Ånestad et al.**
A	A	C	J
B	B	D	K
E	C	G	L
F	D	K	M
H	E	M	N
I	F	O	O
J	G	P	P
L	H		
N	I		

Bioinformatic analysis

Raw reads were adaptor-trimmed and quality-filtered using fastp v. 0.23.2 with the options: -q 20, --detect_adapter_for_pe, -5, -r, -W 4, -M 20 (3). Read quality was assessed with FastQC v. 0.12.1 (4) and summarized using MultiQC v. 1.14 (5). *De novo* genome assemblies were generated using SPAdes v. 4.0.0 (6) with the --isolate and --plasmid flags. Assemblies generated with the --isolate flag were used for downstream analyses, while plasmid-mode assemblies were used exclusively for plasmid detection.

Both assemblies generated in this study and publicly available NCBI assemblies underwent quality control. Assembly correctness, completeness, and contiguity were assessed using BUSCO v. 5.8.2 (7, 8) with the option: --lineage pasteurellales_odb12, and QUAST v. 5.3.0 (9), using PM FDAARGOS_218 (ASM207325v2, Strain: FDAARGOS_218. RefSeq: GCF_002073255.2) as the reference genome. Species identity was confirmed using FastANI v.1.34 (10), with average nucleotide identity (ANI) calculated from genome assemblies, by comparing each isolate assembly to the reference genome as above (ANI threshold ≥95%). Subspecies-level assignment was attempted using FastANI by comparing genome assemblies to the type strains of subspecies *multocida* NCTC 10322 (Accession: LT906458), *gallicida* NCTC 10204 (Accession: LR134298), and *septica* NCTC 11995 (Accession: UBSV00000000). ANI values ≥98% were interpreted as indicative of the same subspecies (11).

As ANI did not reliably distinguish between subspecies *multocida* and *gallicida* due to their high genomic similarity, differentiation between these two subspecies was performed by screening for the presence of the *gatD* gene, which is unique to the *gallicida* strain (12). The gene was considered present when BLASTn results showed ≥ 80% identity and ≥ 50% coverage (alignment length/subject length).

Assemblies passing quality control were annotated using Bakta v. 1.10.3 (13) with default parameters. Figure S1 summarizes the workflow from sample collection up to different steps of bioinformatic analyses.

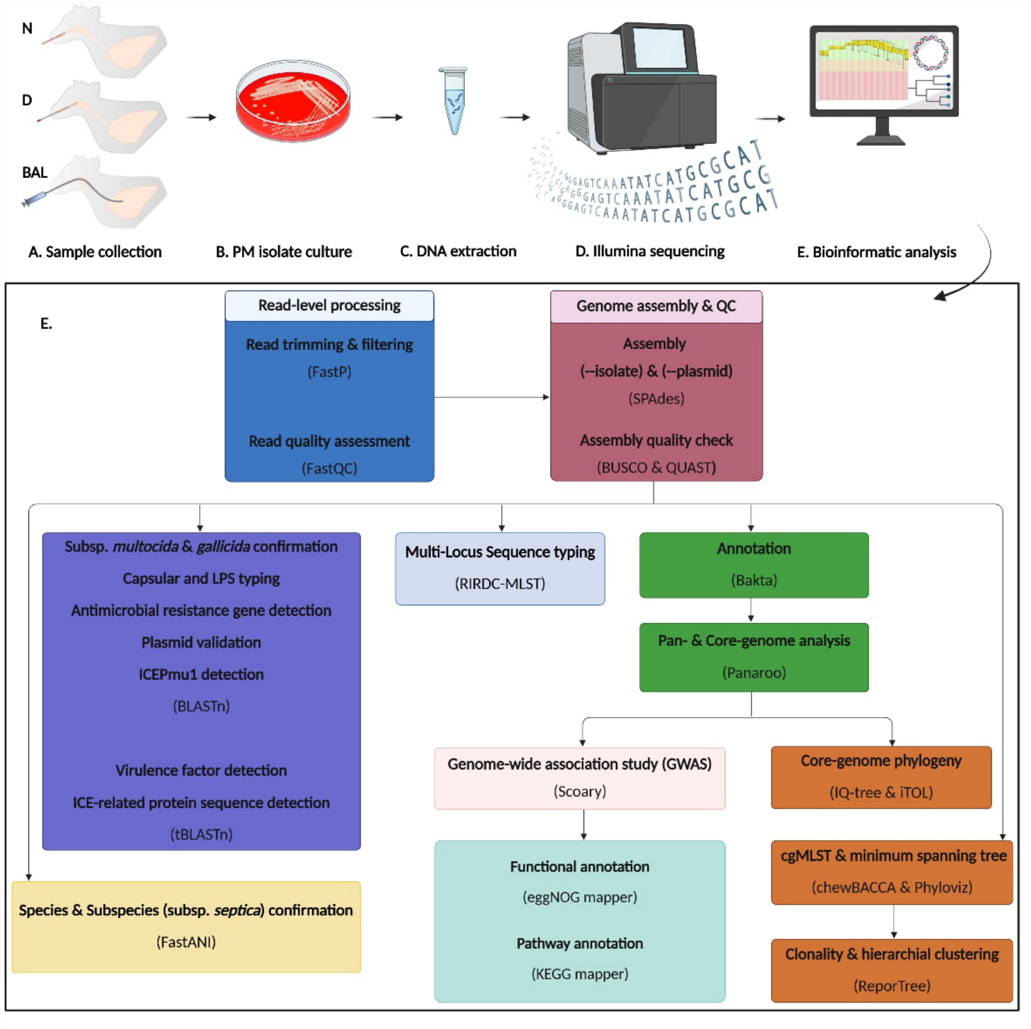

Figure S1. Overview of the workflow from sample collection to bioinformatic analysis of *Pasteurella multocida* isolates. The figure illustrates the process from sample collection (A), culturing of 370 *P. multocida* (PM) isolates (B), followed by DNA extraction (C), whole-genome sequencing (D), and downstream bioinformatic analyses (E), including genome processing, comparative genomics, and association analyses. Panel A was adapted with permission from an illustration by Lise Marie Ånestad (1). All subsequent steps were created using BioRender (Ahmadi, A. (2026), https://BioRender.com/ec2fcl1).

Results

High-quality assemblies of P. multocida subspecies multocida

Of the 370 assemblies, five were excluded because their genome sizes were outside the expected range (2.20 - 2.78 Mb). Tables S2 and S3 summarize the assembly quality control and annotation results, respectively, and include comparisons with NCBI assemblies.

Table S2. Overview of *Pasteurella multocida* genome assembly quality statistics and completeness, assessed by Quast and BUSCO, respectively. For each metric, the upper values indicate the minimum and maximum, and the lower value represents the mean ± SD.

	Quast								BUSCO		
	**# contigs (>500 bp)**	**Length (bp)**	**GC (%)**	**N50**	**L50**	**auN**	**NG50**	**Genome fraction (%)**	**Completeness (%)**	**Fragmented (%)**	**Missing (%)**
**Norway**	16-254	2210120-2532268	40.1-41.4	51134-245460	2-14	62299.1-464513.5	51134-242992	83-89.7	98.1-99.6	0.3-1	0.1-0.8
**(n=365)**	49.9 ± 32	2349114.4 ± 126724	40.2 ± 0.07	156419 ± 63039	6.1 ± 3.2	200251.9 ± 97288.5	144567 ± 44932	88.5 ± 1.8	99.3 ± 0.4	0.5 ± 0.2	0.2 ± 0.2
**NCBI**	1-365	2185210-2699012	39.9-41	10499-2699012	1-68	12221.1-2699012	10069-2699012	82.6-92	87.3-99.7	0.2-3.9	0.1-8.8
**(n=345)**	43.6 ± 44.8	2354570.7 ± 93314.3	40.3 ± 0.1	508533 ± 782865.3	5.6 ± 7.3	538288.6 ± 765817.4	510191 ± 782080.8	89.4 ± 1	99.3 ± 1	0.4 ± 0.4	0.3 ± 0.7

Table S3. Bakta annotation results for Norwegian bovine isolates and publicly available bovine *Pasteurella multocida* genomes retrieved from the NCBI database. For each metric, the upper values indicate the minimum and maximum, and the lower value represents the mean ± SD.

	**CDS**	**Hypothetical**	**Pseudogenes**	**ORFs**	**tRNA**	**rRNA**	**tmRNA**	**ncRNA**
**Norway**	2022 – 2491	39 – 249	3 – 34	1 – 3	43 – 59	4 – 9	1 – 2	12 – 19
**(n=365)**	2239 ± 189	94 ± 53	8 ± 8	2 ± 1	50 ± 4	5 ± 1	1 ± 1	15 ± 2
**NCBI**	1975 – 2610	20 – 216	0 – 40	0 – 3	20 – 67	0 – 19	1 – 1	10 – 20
**(n=345)**	2198 ± 119	72 ± 30	6 ± 6	2 ± 1	52 ± 6	7 ± 5	1 ± 0	14 ± 2

ANI comparisons against the *P. multocida* subsp. *septica* type strain was below 98% for all isolates except one NCBI genome, confirming its classification as subsp. *septica (*Figure S2). In contrast, most isolates showed ≥98% ANI to both subspecies *multocida* and *gallicida*. However, 28 Norwegian isolates had ANI values between 97-98% relative to subsp. *gallicida* (Figure S2), indicating that ANI is insufficient to reliably distinguish between these two subspecies. Therefore, BLASTn analysis targeting the *gatD* gene was performed, confirming four NCBI genomes as subspecies *gallicida*, with the remainder classified as *multocida.* Table S4 summarizes the classification of all assemblies and their genomic profiles.

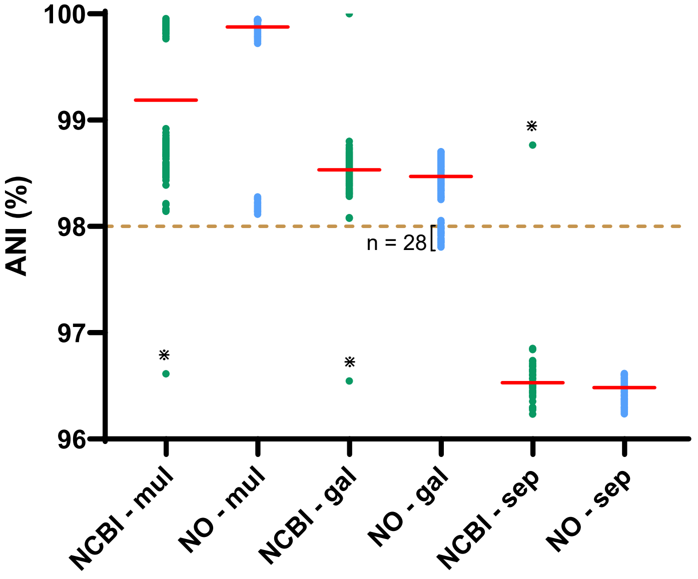

Figure S2. Overview of ANI values for subspecies assignment of Norwegian and NCBI *P. multocida assemblies.* Red lines indicate mean ANI values, and the dashed line represents the 98% ANI threshold used for subspecies assignment. The asterisk denotes the assembly that diverged from subsp. *multocida* and subsp. *gallicida* and was subsequently confirmed as subsp. *septica.* Abbreviations: mul, *multocida*; gal, *gallicida*; sep, septica; NO: Norwegian assemblies.

Table S4. Norwegian and NCBI *P. multocida* genomes classified as subsp. *multocida*, *gallicida*, or *septica*, with corresponding genotypes. Abbreviation: Cap, capsular type; LPS, lipopolysaccharide group; ST, sequence type; ND, not detected.

**Isolates**	**Genotype Cap: LPS: ST**	***P. multocida* subsp. *multocida***	***P. multocida* subsp. *gallicida***	***P. multocida* subsp. *septica***
**GCA_034011055.1**	CapA: LPS1: ST36			✓
**GCA_023555775.1**	CapA: LPS1: ST452		✓	
**GCA_029656225.1**	CapA: ND: ST51		✓	
**GCA_034011735.1**	CapA: LPS1: ST58		✓	
**GCA_900636625.1**	CapA: LPS1: ST65		✓	
**Rest of NCBI assemblies**	340 genomes	✓		
**Norwegian assemblies**	365 isolates	✓		

Mobile genetic elements: Plasmids and integrative and conjugative elements (ICEs)

Generated plasmid assemblies showed no complete plasmid replicons known in PLASDB. Similarly, no ICEPmu1 was detected in the Norwegian isolates.

Search for ICE protein sequences of the ICEberg database in the Norwegian assemblies yielded hits to conserved proteins, which were not co-localized or organized into ICE-like structures (Table S5).

Table S5. ICE-associated proteins identified in *Pasteurella multocida* genome assemblies of Norwegian isolates using tBLASTn.

Detected ICE proteins	ICE Berg ID	Accession no.	No. of isolates	Sequence types
**Single-stranded DNA-binding protein** **[*Pasteurella multocida 36950*]**	ICEberg|440	AET15283.1	14	12 isolates of ST173; ST561
**Single-stranded DNA-binding protein** **[*Histophilus somni 2336*]**	ICEberg|441	ACA31524.1		
**Single-stranded DNA-binding protein Ssb** **[*Mannheimia haemolytica M42548*]**	ICEberg|987	AGK02762.1		
**Peptide chain release factor 3** **[*Vibrio alginolyticus*]**	ICEberg|1040 ICEberg|1043 ICEberg|1046	ALJ83396.1 ALF34863.1 ALF34977.1	365	All STs
**Peptide chain release factor 3** **[*Vibrio fluvialis Ind1*]**	ICEberg|36	ACV96536.1	365	All STs
**Peptide chain release factor 3** **[*Proteus mirabilis HI4320*]**	ICEberg|40	CAR44788.1	365	All STs
**Peptide termination factor** **[*Vibrio cholerae Ban5*]**	ICEberg|12	ACV96186.1	328	All ST except ST173
**Peptide termination factor** **[*Vibrio cholerae MJ-1236*]**	ICEberg|16	ACQ61816.1	365	All STs
**Peptide termination factor** **[*Vibrio cholerae Ind4*]**	ICEberg|21	ACV96297.1	328	All ST except ST173
**Peptide termination factor** **[*Vibrio cholerae Mex1*]**	ICEberg|23	ACV96449.1	328	All ST except ST173
**50S ribosomal protein L34** **[*Alteromonas mediterranea DE1*]**	ICEberg|957	WP_014290886.1	365	All STs
**Peptide chain release factor 3** **[*Alteromonas mediterranea DE1*]**	ICEberg|957	WP_015066603.1	365	All STs
**Peptide chain release factor 3**	ICEberg|958	WP_015066603.1	365	All STs

[***Alteromonas mediterranea UM4b***]				
**Peptide chain release factor 3** [***Alteromonas mediterranea MED64***]	ICEberg|959	WP_015066603.1	365	All STs
**IS5 family element, transposase orfA** [***Bordetella_petrii***]	ICEberg|220	CAP41759.1	1	One isolate of ST79

Virulence factor patterns

Using a customized virulence factor (VF) database adapted from (14, 15) and supplemented with common *P. multocida* VFs retrieved from NCBI and the Virulence Factor Database (VFDB), tBLASTn analysis identified multiple VFs in the assembled genomes (Table S6). Based on presence-absence patterns of VFs in the Norwegian isolates, 13 different virulence profiles (VP) were defined (Table S7).

Table S6. Frequency and proportion (%) of selected virulence factors detected by tBLASTn in Norwegian (n = 365) and publicly available bovine *Pasteurella multocida* genomes (NCBI, n=345).

Description	Protein	Accession. No.	Number of isolates (%)	
			**Norway**	**NCBI**
Hemoglobin-binding protein	**HgbA**	AAQ14873.1	365 (100%)	340 (98.5%)
	**HgbB (partial)**	APX53047.1	37 (10.1%)	135 (39.1%)
	**HgbB_PM0337**	AAK02421.1	0 (0%)	0 (0%)
Outer membrane family protein	**OmpA**	WP_005726415.1	365 (100%)	343 (99.4%)
	**Omp16**	AHW46109.1	365 (100%)	345 (100%)
	**Oma87**	EPE72935.1	365 (100%)	345 (100%)
	**OmpH-1**	SNV59447.1	173 (47.4%)	189 (54.8%)
	**OmpH-2**	VEE38334.1	365 (100%)	343 (99.4%)
	**OmpH-3**	AMK08231.1	365 (100%)	259 (75%)
Transferrin binding protein A	**TbpA**	AAK02460.1	365 (100%)	344 (99.7%)
Pili or (fimbrial) subunit protein A	**PtfA**	AKO69808.1	365 (100%)	272 (78.8%)
Competence protein E	**ComE**	AFF25459.1	279 (76.4%)	339 (98.3%)
Protective outer membrane lipoprotein	**PlpE**	AAK03601.1	328 (89.9%)	237 (68.7%)
	**PlpP**	AAK03602.1	328 (89.9%)	320 (92.7%)
	**PlpB**	AAK03814.1	365 (100%)	345 (100%)
	**PlpD**	VEE38139.1	365 (100%)	343 (99.4%)
Filamentous hemagglutinin protein	**PfhB1**	AAK02141.1	328 (89.9%)	178 (51.6%)
	**PfhB2**	AAK02143.1	300 (82.2%)	305 (88.4%)
putative filamentous hemagglutinin (PmFHA)	**PfhA**	AAK61595.1	328 (89.9%)	317 (91.9%)
putative immunoglobulin binding protein (IgBP)		AAK61596.1	328 (89.9%)	235 (68.1%)
Hyaluronan synthase	**PmHAS**	AAC38318.2	365 (100%)	255 (73.9%)
Auto-transporter adhesin family	**Yersinia adhesin-like protein (YadA-1)**	WP_221812505.1	300 (82.2%)	153 (44.3%)
	**Yersinia adhesin-like protein (YadA-2)**	WP_015702581.1	328 (89.9%)	158 (45.8%)

	**ESPR-type extended signal peptide-containing protein**	WP_075270724.1	300 (82.2%)	230 (66.7%)
	**High-molecular-weight Surface Fibril (Hsf-1)**	AAK02798.1	0 (0%)	86 (24.9%)
	**High-molecular-weight Surface Fibril (Hsf-2)**	ANJ91000.1	328 (89.9%)	158 (45.8%)
Exo-alpha-sialidase H	**NanH**	WP_005756716.1	328 (89.9%)	332 (96.2%)
Sialidase B	**NanB**	APW54750.1	37 (10.1%)	95 (27.5%)
N-acylneuraminate cytidylyltransferase	**NeuA**	WP_005723432.1	365 (100%)	344 (99.7%)
Superoxide dismutase	**SodA**	WP_005750998.1	365 (100%)	345 (100%)
	**SodC**	WP_005725042.1	365 (100%)	345 (100%)
Energy transducer TonB	**TonB**	WP_005757324.1	365 (100%)	345 (100%)
TonB-system energizer ExbB	**ExbB**	WP_005717562.1	365 (100%)	345 (100%)
TonB system transport protein ExbD	**ExbD**	WP_005723568.1	365 (100%)	345 (100%)
TonB-dependent heme/hemoglobin acquisition system receptor	**HasR**	AAK03706.1	362 (99.2%)	338 (98%)
TonB-dependent heme/hemoglobin receptor	**HemR**	AAK02660.1	365 (100%)	256 (74.2%)
TonB-dependent receptor protein	**TonBDR-A**	WP_005757608.1	365 (100%)	345 (100%)
TonB-dependent receptor protein domain-containing protein	**TonBDR-B**	WP_032854183.1	328 (89.9%)	247 (71.6%)
	**TonBDR-C**	WP_005756831.1	325 (89%)	156 (45.2%)
TonB-dependent hemoglobin/transferrin/ lactoferrin family receptor	**TonBD-HTLR**	WP_005756819.1	328 (89.9%)	339 (98.3%)
TonBDR plug domain-containing protein	**TonBDR-PD-A**	WP_195188888.1	37 (10.1%)	99 (28.7%)
	**TonBDR-PD-B**	WP_208933817.1	328 (89.9%)	246 (71.3%)
	**TonBDR-PD-C**	WP_306610798.1	365 (100%)	340 (98.5%)
Ferric iron uptake transcriptional regulator	**Fur**	WP_005721511.1	365 (100%)	345 (100%)
Fimbrillin protein	**FimA**	AAK03010.1	0 (0%)	17 (4.9%)
Zinc ABC transporter substrate-binding protein	**ZnuA**	WP_014667908.1	328 (89.9%)	229 (66.4%)
Fimbrial low-molecular-weight protein, pilus assembly system	**Flp1**	SNV65669.1	328 (89.9%)	240 (69.6%)
Tight adherence, Tetratricopeptide repeat protein	**TadD**	WP_014667937.1	328 (89.9%)	173 (50.1%)
Dermonecrotic toxin ToxA	**ToxA**	WP_015691094.1	0 (0%)	5 (1.4%)
Elongation factor Tu [*Francisella tularensis*]	**Tuf**	WP_003028672.1	266 (72.9%)	320 (92.7%)
bifunctional D-glycero-beta-D-manno-heptose-7-phosphate kinase/D-glycero-beta-D-manno-heptose 1-phosphate adenylyltransferase HldE [*Haemophilus influenzae*]	**HldE**	WP_005693548.1	365 (100%)	345 (100%)
ADP-glyceromanno-heptose 6-epimerase [*Haemophilus*]	**RfaD**	WP_005632797.1	365 (100%)	344 (99.7%)
RdgB/HAM1 family non-canonical purine NTP pyrophosphatase [*Haemophilus influenzae*]	**RdgB**	WP_005694045.1	365 (100%)	345 (100%)
UDP-glucose 4-epimerase GalE [*Haemophilus influenzae*]	**GalE**	WP_005694325.1	365 (100%)	343 (99.4%)
UTP-glucose-1-phosphate uridylyltransferase GalU [*Haemophilus influenzae*]	**GalU**	WP_005693178.1	365 (100%)	344 (99.7%)
D-sedoheptulose 7-phosphate isomerase [*Haemophilus influenzae*]	**LpcA**	WP_005694260.1	365 (100%)	345 (100%)

lipid-A-disaccharide synthase [*Haemophilus influenzae*]	**LpxB**	WP_010869121.1	328 (89.9%)	246 (71.3%)
UDP-3-O-acyl-N-acetylglucosamine deacetylase [*Haemophilus influenzae*]	**LpxC**	WP_005693459.1	365 (100%)	345 (100%)
UDP-3-O-(3-hydroxymyristoyl) glucosamine N-acyltransferase [*Haemophilus influenzae*]	**LpxD**	WP_005693262.1	365 (100%)	344 (99.7%)
lipopolysaccharide heptosyltransferase II [*Haemophilus influenzae*]	**WaaF**	WP_005693429.1	365 (100%)	345 (100%)
3-deoxy-8-phosphooctulonate synthase [*Haemophilus influenzae*]	**KdsA**	WP_005693586.1	365 (100%)	345 (100%)
UDP-N-acetylglucosamine--undecaprenyl-phosphate N-acetylglucosaminephosphotransferase [*Haemophilus influenzae*]	**wecA**	WP_005694200.1	363 (99.4%)	344 (99.7%)
KpsF/GutQ family sugar isomerase [*Haemophilus influenzae*]	**KpsF**	WP_032828451.1	0 (0%)	187 (54.2%)
Adenylate cyclase [*Pseudomonas aeruginosa PAO1*]	**ExoY**	NP_250881.1	0	1
ABC transporter ATP-binding protein [*Neisseria meningitidis*]	**TagH**	WP_002224750.1	0	1
Heptosyltransferase II [*Pseudomonas aeruginosa PAO1*]	**WaaF-2**	NP_253699.1	0	1

Table S7. Variable virulence factors (VFs) identified in Norwegian *P. multocida* isolates. VFs present in all or none of the isolates were excluded. Presence or absence of each VF is indicated by "+" and "–", respectively. Based on VF presence-absence patterns, 13 distinct virulence profiles (VPs) were defined. VFs sharing identical presence-absence profiles across isolates were grouped into virulence groups (VGs) to reduce redundancy in reporting. These groups are defined as follows: ***VG1:** nanH, PmFHA, IgBP, PfhB1, PlpE, PlpP, YadA-2, Hsf-2, TadD, ZnuA, Flp1, TonBD-HTLR, TonBDR-B, TonBDR-PD-B, and LpxB; ****VG2:** HgbB, nanB, and TonBDR-PD-A; *****VG3:** YadA-1 and ESPR.

STs	VP	VG1*	VG2**	VG3***	ComE	Omp-H1	PfhB2	TonBDR-C	TuF	WecA	Total No. isolates
**ST13**	**1**	+	-	+	+	-	+	+	-	+	17
	**2**	+	-	+	+	-	+	+	+	+	1
	**3**	+	-	-	+	-	+	+	-	+	1
	**4**	+	-	-	+	-	-	-	-	+	3
**ST79**	**5**	+	-	+	+	+	+	+	+	+	128
	**6**	+	-	+	+	+	-	+	+	+	25
	**7**	+	-	+	+	+	+	+	-	+	20
**ST80**	**2**	+	-	+	+	-	+	+	+	+	76
	**1**	+	-	+	+	-	+	+	-	+	6
**ST173**	**8**	-	+	-	-	-	-	-	+	+	18
	**9**	-	+	-	-	-	-	-	-	+	17
	**10**	-	+	-	-	-	-	-	-	-	2
**ST560**	**11**	+	-	-	-	-	+	+	-	+	19
**ST561**	**1**	+	-	+	+	-	+	+	-	+	2

**ST562**	**12**	+	-	+	-	-	+	+	+	+	18
	**13**	+	-	+	-	-	+	+	-	+	7
**ST563**	**11**	+	-	-	-	-	+	+	-	+	5
**No. isolates**		328	37	300	279	173	300	325	266	363	365

Minimum spanning tree of P. multocida isolates in the global context

Allelic differences among Norwegian isolates were calculated using the goeBURST algorithm implemented in Phyloviz. Table S8 summarizes allelic differences between and within STs, and Table S9 shows inter-herd differences among isolates sharing the same ST.

The wgMLST schema was generated using combined Norwegian and NCBI assemblies (Norwgian-NCBI-Dataset 2; n = 710) and comprised 4,207 loci. Allele calling identified 39,947 novel alleles, expanding the schema to 44,154 alleles. 30 paralogous loci were removed. Core genome loci present in ≥95% of genomes defined a cgMLST schema of 1,523 loci.

A total of 181 isolates sharing identical cgMLST95 profiles were excluded from the minimum spanning tree (MST), resulting in 529 non-redundant isolates in the final MST (Figure S3). The polyphyletic pattern of ST13 observed in the core-genome phylogeny (iTOL) was also reflected in the MST, confirming that ST13 does not represent a monophyletic lineage (Figure S3).

Table S8. Allelic differences between (blue) and within (cream) STs of Norwegian *P. multocida* isolates based on cgMLST95, calculated using the goeBURST algorithm. Rows indicate minimum-maximum allelic differences (first), mean ± SD (second), and median (third) differences.

	ST13	ST79	ST80	ST173	ST560	ST561	ST562	ST563
**ST13 (15 isolates)**	1-182<br>67.2 ± 60.9<br>109							
**ST79 (84 isolates)**	229-305<br>259.8 ± 14<br>256	1-68<br>38.1 ± 13.9<br>40						
**ST80 (54 isolates)**	291-334<br>308.8 ± 8.7<br>307	296-328<br>308.7 ± 4.7<br>309	1-60<br>35.4 ± 17.7<br>42					
**ST173 (27 isolates)**	1465-1554<br>1526.9 ± 17.7<br>1539	1531-1554<br>1545.2 ± 8.9<br>1550	1526-1548<br>1539.8 ± 8.9<br>1545	1-153<br>87.9 ± 62.9<br>131				
**ST560 (13 isolates)**	73-138<br>94.8 ± 15.3<br>93	249-272<br>259.8 ± 3.9<br>260	293-318<br>304.1 ± 4.3<br>304	1491-1515<br>1507.5 ± 9.9<br>1514	1-35<br>20.3 ± 12<br>27			
**ST561 (One isolate)**	87-122<br>92.2 ± 10.5<br>88	228-247<br>233.5 ± 3.7<br>233	287-303<br>294 ± 4<br>294	1519-1540<br>1533 ± 9.3<br>1539	82-86<br>83.6 ± 1.3<br>83	0		
**ST562 (17 isolates)**	124-174<br>133.6 ± 12.5<br>127	234-249<br>240.2 ± 2.8<br>240	265-285<br>273.5 ± 3.9<br>274	1525-1545<br>1538 ± 8.5<br>1543	119-130<br>123.1 ± 2.3<br>122	119-123<br>120.5 ± 1.1<br>120	1-10<br>4.5 ± 2.1<br>4	
**ST563 (Five isolates)**	78-139<br>97.6 ± 15.4<br>96	255-271<br>260.8 ± 3.3<br>261	299-318<br>306.9 ± 3.9<br>307	1492-1515<br>1507.6 ± 10.2<br>1514	16-34<br>25.5 ± 6.7<br>23	87-88<br>87.2 ± 0.4<br>87	124-129<br>125.7 ± 1.2<br>125	1-4<br>2.4 ± 1<br>2

Table S9. Inter-herd genetic variation of *P. multocida* isolates within each sequence type (ST) based on cgMLST95 alleles, calculated using the goeBURST algorithm. Values show minimum-maximum allelic differences, mean ± SD (upper row), and median (lower row) differences. ST561, ST562, and ST563 were each observed in a single herd; their intra-ST allelic differences are presented in Table S8.

Sequence types	Minimum-Maximum allele difference between two herds	Mean ± SD<br>Median values of all inter-herd differences within an ST cluster
**ST13 (4 herds)**	108-182	121.8 ± 15.6<br>110
**ST79 (11 herds)**	22-68	42.4 ± 7.1<br>42
**ST80 (5 herds)**	14-60	44.6 ± 5.7<br>43
**ST173 (4 herds)**	18-153	125.6 ± 37.1<br>138
**ST560 (4 herds)**	7-35	26.9 ± 6.4<br>29

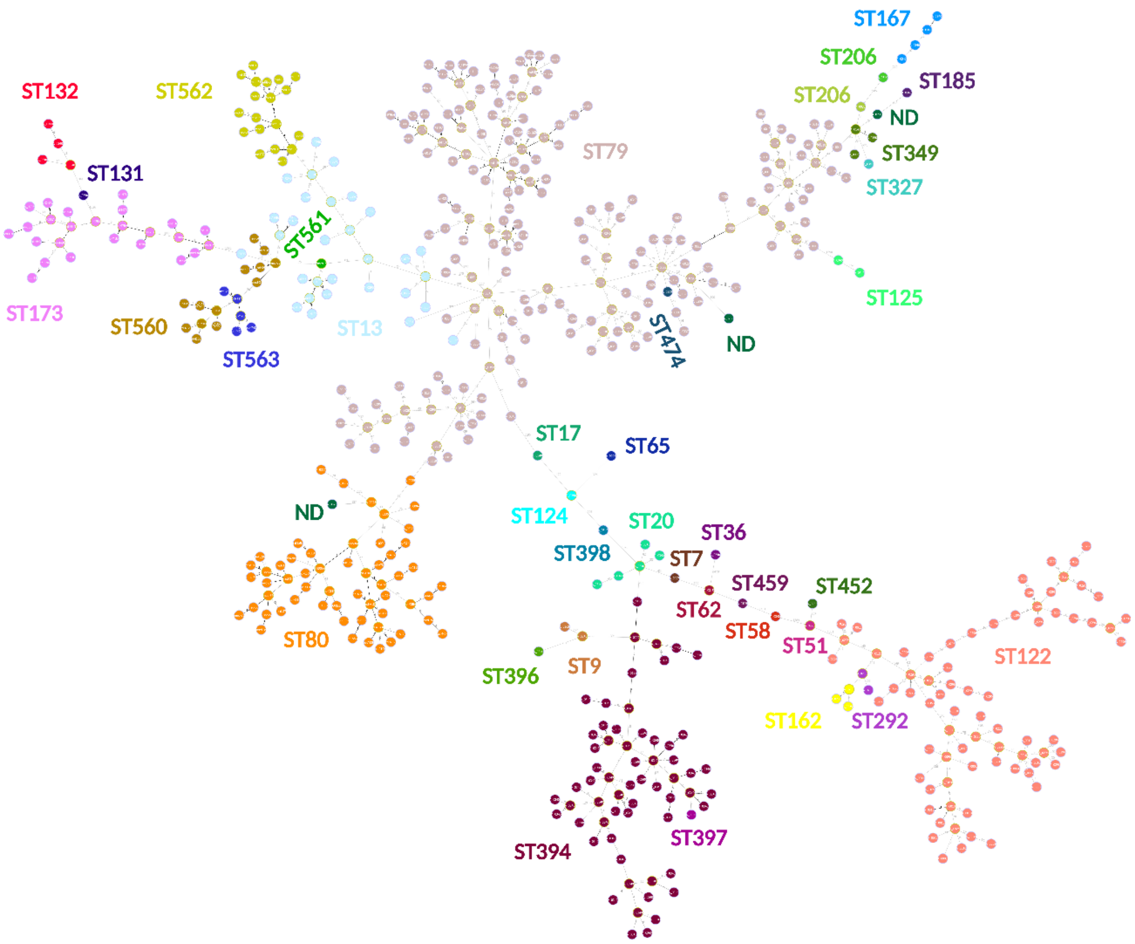

Figure S3. Minimum spanning tree (MST) of combined Norwegian and NCBI *P. multocida* assemblies based on 529 non-redundant cgMLST95 profiles, generated using the goeBURST algorithm in Phyloviz v. 2. Sequence types (ST) are indicated by colours and labels. ND: not detected

Pan-genome of Norwegian-NCBI derived P. multocida genomes

The pan- and core-genomes of Norwegian-NCBI-derived *P. multocida* isolates (n=710) were constructed using Panaroo. In total, 5,560 genes were identified, constituting the species pan-genome. Of this, 1,623 were classified as hard-core genes (present in 99–100% of isolates), 88 as soft-core genes (95–99%), 829 as shell genes (15–95%), and 3,020 as cloud genes (0–15%). The distribution of core and accessory genes across isolates is shown in Figure S4.

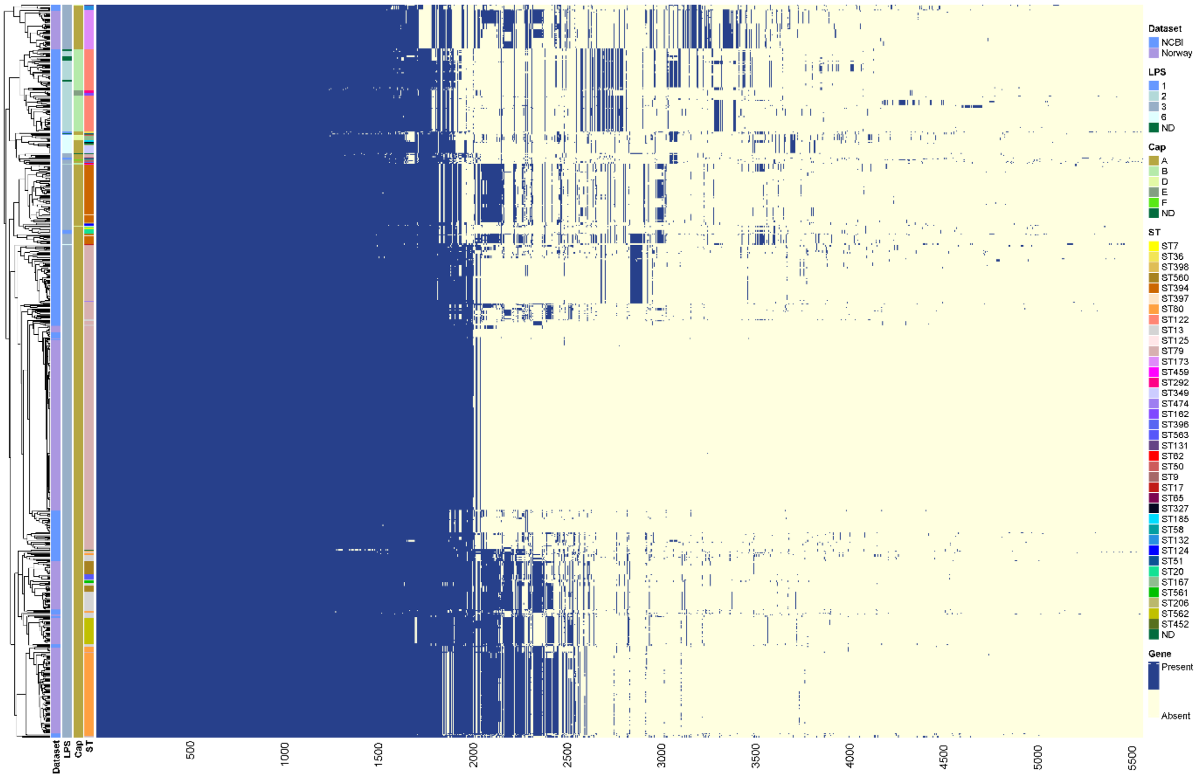

Figure S4. Gene presence–absence heatmap illustrating the pan-genome of 710 Norwegian and NCBI-derived *P. multocida* genomes (PM). Metadata tracks indicate capsular types (Cap), lipopolysaccharide groups (LPS), and sequence types (ST). The dataset comprises 365 PM Norwegian PM isolates and 345 publicly available bovine PM assemblies from NCBI.

References

1. Ånestad LM, Falkeid SE, Oma VS, Garmo RT, Bjelland AM, Woolums AR, et al. Cross-sectional study of calves from Norwegian dairy herds with enzootic pneumonia: pathogen occurrence, antimicrobial resistance, culture result interpretation, and sampling site agreement. BMC Vet Res. 2026.
2. Klem TB, Sjurseth SK, Sviland S, Gjerset B, Myrmel M, Stokstad M. Bovine respiratory syncytial virus in experimentally exposed and rechallenged calves; viral shedding related to clinical signs and the potential for transmission. BMC Vet Res. 2019;15(1):156.
3. Chen S, Zhou Y, Chen Y, Gu J. fastp: an ultra-fast all-in-one FASTQ preprocessor. Bioinformatics. 2018;34(17):i884–i90.
4. Andrews S. FastQC: A Quality Control Tool for High Throughput Sequence Data 2010 [Available from: http://www.bioinformatics.babraham.ac.uk/projects/fastqc/.
5. Ewels P, Magnusson M, Lundin S, Käller M. MultiQC: summarize analysis results for multiple tools and samples in a single report. Bioinformatics. 2016;32(19):3047–8.
6. Prjibelski A, Antipov D, Meleshko D, Lapidus A, Korobeynikov A. Using SPAdes De Novo Assembler. Current Protocols in Bioinformatics. 2020;70(1):e102.
7. Manni M, Berkeley MR, Seppey M, Zdobnov EM. BUSCO: Assessing Genomic Data Quality and Beyond. Curr Protoc. 2021;1(12):e323.
8. Manni M, Berkeley MR, Seppey M, Simão FA, Zdobnov EM. BUSCO Update: Novel and Streamlined Workflows along with Broader and Deeper Phylogenetic Coverage for Scoring of

Eukaryotic, Prokaryotic, and Viral Genomes. Molecular Biology and Evolution. 2021;38(10):4647–54.
9. Gurevich A, Saveliev V, Vyahhi N, Tesler G. QUAST: quality assessment tool for genome assemblies. Bioinformatics. 2013;29(8):1072–5.
10. Jain C, Rodriguez RL, Phillippy AM, Konstantinidis KT, Aluru S. High throughput ANI analysis of 90K prokaryotic genomes reveals clear species boundaries. Nat Commun. 2018;9(1):5114.
11. Smallman TR, Perlaza-Jiménez L, Wang X, Korman TM, Kotsanas D, Gibson JS, et al. Pathogenomic analysis and characterization of Pasteurella multocida strains recovered from human infections. Microbiology Spectrum. 2024;12(4):e03805–23.
12. Ujvári B, Gantelet H, Magyar T. Development of a multiplex PCR assay for the detection of key genes associated with Pasteurella multocida subspecies. Journal of Veterinary Diagnostic Investigation. 2022;34(2):319–22.
13. Schwengers O, Jelonek L, Dieckmann MA, Beyvers S, Blom J, Goesmann A. Bakta: rapid and standardized annotation of bacterial genomes via alignment-free sequence identification. Microbial Genomics. 2021;7(11).
14. Smith E, Miller E, Aguayo JM, Figueroa CF, Nezworski J, Studniski M, et al. Genomic diversity and molecular epidemiology of Pasteurella multocida. PLoS One. 2021;16(4):e0249138.
15. Emily S. Pmul-databases 2021 [Available from: https://github.com/JohnsonSingerLab/Pmult-databases.
